# Reductive Hydroformylation of Isosorbide Diallyl Ether

**DOI:** 10.3390/molecules26237322

**Published:** 2021-12-02

**Authors:** Jérémy Ternel, Adrien Lopes, Mathieu Sauthier, Clothilde Buffe, Vincent Wiatz, Hervé Bricout, Sébastien Tilloy, Eric Monflier

**Affiliations:** 1 University of Artois, CNRS, Centrale Lille, University of Lille, UMR 8181–UCCS–Unité de Catalyse et Chimie du Solide, F-62300 Lens, France; jeremy.ternel@univ-artois.fr (J.T.); adrien.figueiredo.lopes@gmail.com (A.L.); herve.bricout@univ-artois.fr (H.B.); eric.monflier@univ-artois.fr (E.M.); 2 University of Lille, CNRS, Centrale Lille, ENSCL, University of Artois, UMR 8181, Unité de Catalyse et Chimie du Solide, F-59000 Lille, France; mathieu.sauthier@univ-lille.fr; 3 Roquette Frères, 1 Rue de la Haute Loge, F-62136 Lestrem, France; clothilde.buffe@roquette.com (C.B.); vincent.wiatz@roquette.com (V.W.)

**Keywords:** catalysis, hydroformylation, hydrogenation, rhodium, tandem reaction

## Abstract

Isosorbide and its functionalized derivatives have numerous applications as bio-sourced building blocks. In this context, the synthesis of diols from isosorbide diallyl ether by hydrohydroxymethylation reaction is of extreme interest. This hydrohydroxymethylation, which consists of carbon-carbon double bonds converting into primary alcohol functions, can be obtained by a hydroformylation reaction followed by a hydrogenation reaction. In this study, reductive hydroformylation was achieved using isosorbide diallyl ether as a substrate in a rhodium/amine catalytic system. The highest yield in bis-primary alcohols obtained was equal to 79%.

## 1. Introduction

The depletion of fossil resources is a growing problem. Consequently, the challenge is to find renewable substitutable resources for the synthesis of chemical products. So, in the context of green chemistry and sustainable development, the synthesis of simple molecules from biomass with chemically transformable functions is an essential goal. Moreover, functionalization, such as the use of highly selective catalytic processes with a high economy of atoms, must be in line with the principles of green chemistry. The polymer industry represents a large part of the world fossil resource consumption. In this sense, the design of new monomers from bio-based molecules represents a major challenge concerning the reduction of fossil resources. Biomass is mainly composed of vegetable oils (5–13%), lignin (15–25%), and carbohydrates (61–82%) [1]. The valorization of this last segment, mainly present in biomass, makes it possible to obtain polyfunctional molecules such as furans, lactic acid, and, more particularly, sorbitol obtained from glucose, which can be accessible from starch. Sorbitol and its isomers can be dehydrated to form dianhydrohexitols that bear bicyclic scaffolds and secondary diols. Among the possible isomers, isosorbide is produced on an industrial scale, with the Roquette company being one of the largest producers. The literature describes numerous bio-sourced compounds accessible from isosorbide, which have multiple applications, such as in the medical field for isosorbide dinitrates [2,3], in non-toxic green solvents for dimethyl isosorbide [4], and, more particularly, in polymer chemistry. Many reviews list a large number of biopolymers derived from isosorbide or its functionalized derivatives [5,6,7,8]. Isosorbide is mainly employed as a monomer in the production of polyesters and polycarbonates [9,10,11,12]. Isosorbide derivatives, where the hydroxyl groups are replaced by amine, carboxylic acid, or nitrile functions are described [13,14,15]. Other derivatives, where the reactive function or a carbon-carbon double bond are carried by an extra-cyclic carbon, have also been synthesized. More particularly, the synthesis of isosorbide diallyl ether has been extensively studied for its transformation into isosorbide bis-glycidyl ether (Bisglycidyl Ether) by epoxidation of the double bonds [16]. Although the isosorbide bis-glycidyl ether is often described in the literature, little attention has been paid to other possible transformations of the isosorbide diallyl ether derivative by functionalization of the carbon-carbon double bonds. More precisely, organometallic catalysis offers many opportunities for incorporating polymerisable functions such as carboxylic acid, ester, aldehyde, amine, or alcohol on allyl derivatives of isosorbide. In this context, we were interested in the synthesis of diols from isosorbide diallyl ether by hydrohydroxymethylation (HHM) reaction. HHM consists of the conversion of carbon-carbon double bonds into primary alcohol functions via a hydroformylation reaction, followed by a hydrogenation reaction [17]. These primary alcohol functions can be produced in one- or two-step processes by using one or two catalysts, and under similar or different reaction conditions [18,19,20,21]. The process in a one-step reaction with a single catalyst is the best alternative, owing to the reduction in the amount of the catalyst, the reaction time, and the overall cost. In this context, one of the best systems is a Rh-catalyst associated with tertiary amines in the presence of CO/H_2_ [22,23,24,25,26,27,28,29,30,31,32,33]. In this publication, we report the HHM of isosorbide diallyl ether using this catalytic system. The effects of various reaction parameters (nature and concentration of amines, time reaction, and syngas composition) were studied in order to increase the production of primary alcohols.

## 2. Results

### 2.1. Presentation of the Compounds Resulting from HHM of IDE

**Scheme 1:**
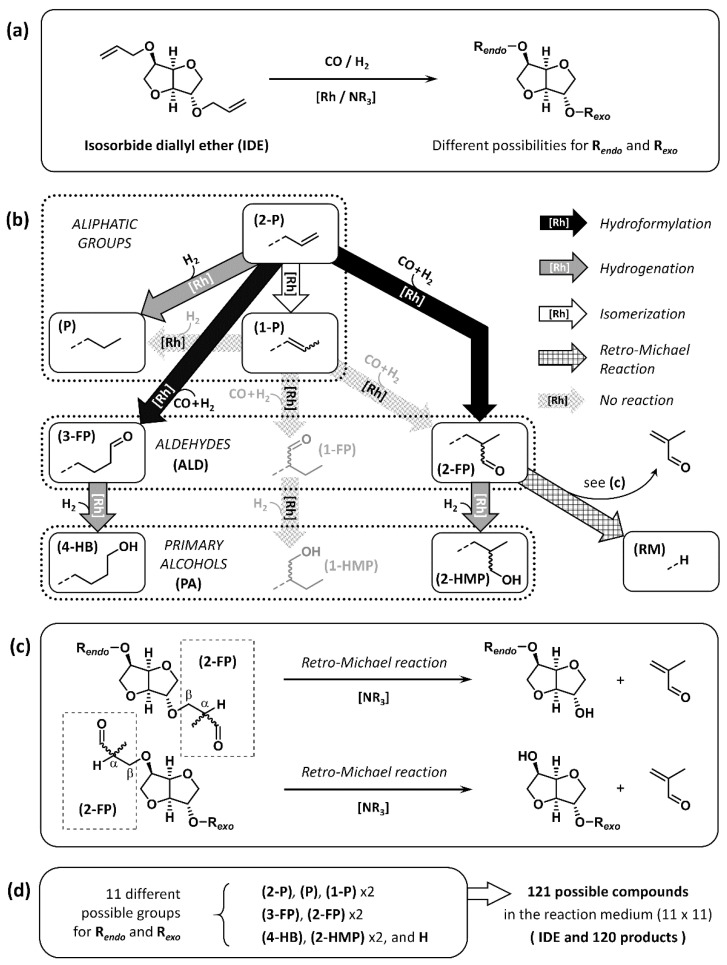
Reactions of isosorbide diallyl ether (IDE) in the presence of the [Rh(acac)(CO)_2_/NR_3_] catalytic system and under CO/H_2_ pressure. (**a**) General equation; (**b**) different possible groups linked to the isosorbide moiety; (**c**) two possible retro-Michael reactions; and (**d**) determination of the total number of possible compounds in the reaction medium. The various groups are allyl **(2-P)**, propyl **(P)**, *(Z)*- or *(E)*-1-propenyl **(1-P),** 3-formylpropyl **(3-FP)**, *(R)-* or *(S)-*2-formylpropyl **(2-FP)**, 4-hydroxybutyl **(4-HB)**, *(R)-* or *(S)-*2-(hydroxymethyl)propyl **(2-HMP)**, and a hydrogen atom **(RM)**. **(1-FP)** and **(1-HMP)** groups are not formed because **(1-P)** group was proven to be unreactive in our experimental conditions (see Scheme 2).

The isosorbide diallyl ether (IDE) used in the study was synthesized by the reaction of isosorbide with allyl bromide in a basic aqueous medium in a similar fashion to previous publications [34,35]. The hydrohydroxymethylation of isosorbide diallyl ether (IDE) by the [Rh(acac)(CO)_2_/amine] catalytic system under CO/H_2_ pressure can produce many compounds. Scheme 1 summarized the theoretical compounds. In particular, isosorbide diallylic ether was expected to be hydrohydroxymethylated into bis-primary alcohols. However, due to the nature of the allylated isosorbide substrate and the isomerizating, hydroformylating, and hydrogenating properties of the [Rh(acac)(CO)_2_/amine] catalytic system under CO/H_2_ pressure, many products could be obtained under catalytic conditions (Scheme 1a). More precisely, each allyl group (2-propenyl group **(2-P)**) of the allylated isosorbide could be converted into a propyl group **(P)** by hydrogenation of the carbon-carbon double bond, or isomerized into a *(Z)*- or an *(E)*-1-propenyl group **(1-P)** (Scheme 1b).

**Scheme 2:**
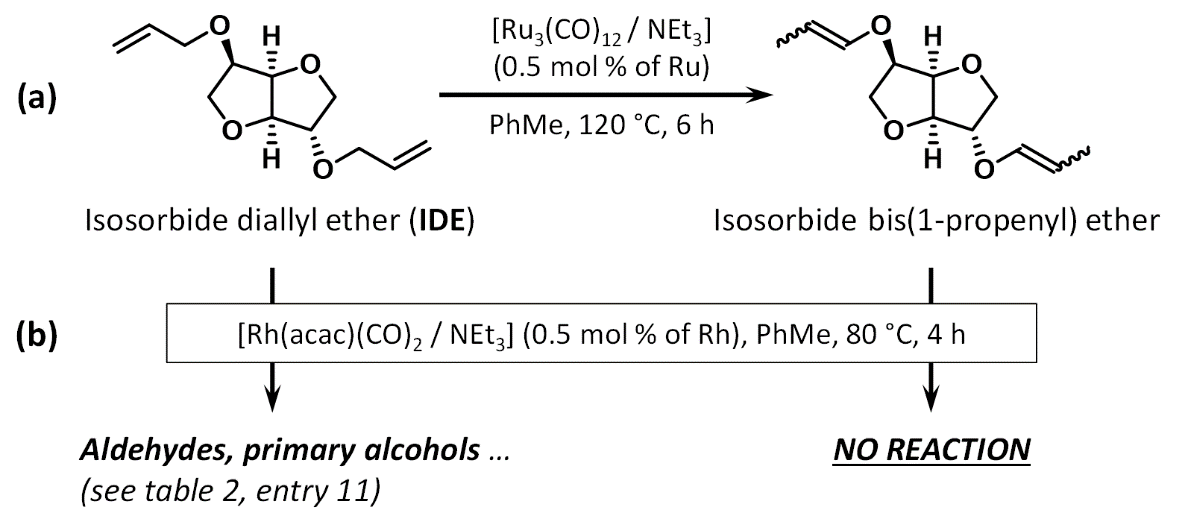
(**a**) Synthesis of isosorbide bis(1-propenyl) ether from isosorbide diallyl ether and (**b**) comparison of these two compounds behaviors under rhodium-catalyzed hydrohydroxymethylation conditions.

Because this **(1-P)** group proved to be completely unreactive in our experimental conditions (see Section 2.2 and Scheme 2), its transformation into **(P)** group by hydrogenation and to 1-formylpropyl **(1-FP)** and 2-formylpropyl **(2-FP)** groups by hydroformylation was excluded. In the same way, because the **(1-FP)** group was not formed, its 1-(hydroxymethyl)propyl **(1-HMP)** corresponding hydrogenated form was also not taken into account.

On the other hand, each allyl **(2-P)** group could be hydroformylated into a linear or branched aldehyde graft, namely, 3-formylpropyl **(3-FP)** and 2-formylpropyl **(2-FP)** groups. The two aldehyde grafts **(3-FP)** and **(2-FP)** (**(ALD)** for the general term) could then be hydrogenated into 4-hydroxybutyl **(4-HB)** and 2-(hydroxymethyl)propyl **(2-HMP)** primary alcohol groups, respectively (**(PA)** for the general term). Furthermore, the aldehyde function of the **(2-FP)** graft showed a hydrogen atom and an oxygen atom on the α and β positions, respectively (Scheme 1c). So, in the presence of the basic amine ligand, a retro-Michael reaction could not be excluded [36]. This reaction would lead to the cleavage of the **(2-FP)** graft with the formation of 2-methylacrolein and the initial ether-oxide function would be transformed into a secondary alcohol function on the isosorbide moiety (Scheme 1b,c).

In other words, the **(2-FP)** graft was replaced by a hydrogen atom (named **(RM)** for retro-Michael). Because two stereochemical forms were possible for the **(1-P)** graft (*Z* and *E*), as for **(2-FP)** and **(2-HMP)** grafts (*R* and *S*), 11 different substituents, bearing by the isosorbide moiety, were possible (Scheme 1d): 4 aliphatic groups (**(2-P), (P)**, *(Z)*-**(1-P)** and *(E)*-**(1-P)**), 3 aldehyde groups (**(ALD)** = **(3-FP)**, *(R)*-**(2-FP)**, *(S)*-**(2-FP)**), 3 primary alcohol groups (**(PA)** = **(4-HB)**, *(R)*-**(2-HMP)**, *(S)*-**(2-HMP)**), and the hydrogen atom coming from the retro-Michael reaction (**(RM)**). Because of these 11 possibilities for **R_endo_** and **R_exo_** groups on the isosorbide moiety, 121 compounds could be present in the reaction medium (11 × 11), including the IDE substrate and 120 reaction products (Scheme 1d).

### 2.2. Preliminary Reactions

In order to illustrate the proof of the concept presented earlier, some exploratory experiments were performed. The applied experimental conditions were inspirited by our previous works for HHM of triglycerides [31]. More precisely, the reaction was carried out by using Rh(acac)(CO)_2_ (0.5 mol%) as a rhodium precursor, triethylamine (TEA, 200 eq.) as ligand, and toluene (6 mL) as a solvent under 80 bars of CO/H_2_ at 80 °C during 4 h. For these preliminary reactions, two substrates were envisaged: isosorbide diallyl ether (IDE, isosorbide bearing **(2-P)** groups) and isosorbide bis(1-propenyl) ether (isosorbide bearing **(1-P)** groups). This last substrate was prepared from IDE by using a Ru/TEA catalytic system to isomerize the carbon-carbon double bonds (see Scheme 2; see also part III.2 of Supplementary Materials for exact experimental conditions).

As previously explained, no conversion of the 1-propenyl groups (**(1-P)**) of the isosorbide bis(1-propenyl) ether was observed under HHM reaction conditions. This result was very interesting because it proved that the **(1-FP)** and **(1-HMP)** grafts, potentially formed from the allyl group after isomerization into **(1-P)** (see Scheme 1), would not appear in the reactions involving IDE as substrate.

**Figure 1:**
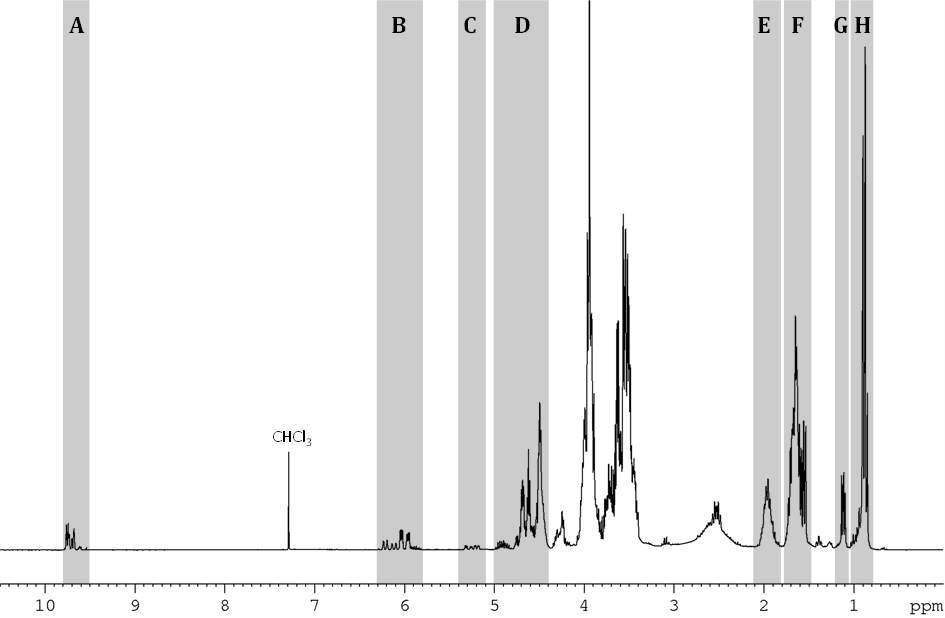
^1^H NMR spectrum of the final HHM reaction medium of isosorbide diallyl ether (IDE) after evaporation of triethylamine and toluene (300 MHz, CDCl_3_, 25 °C); experimental conditions: Rh(acac)(CO)_2_ (12.9 mg, 50 μmol, 1 equiv), TEA (200 equiv), IDE (2.26 g, 10 mmol, 200 equiv), toluene (6 mL), 80 bars of CO/H_2_ (1:1), 80 °C, 4 h; the values of integration of the different chemical shift zones (zones **A** to **H**) were used to determine conversion and yields (see part IV.2 of Supplementary Materials).

In the case of IDE as substrate, the application of the same HHM reaction conditions used for unreactive isosorbide bis(1-propenyl) ether led to numerous products. Figure 1 presents the ^1^H NMR spectrum of the mixture of products obtained after 4 h of reaction (this experiment corresponds to entry 11 of Table 1). The different characteristic ^1^H NMR signals were identified by comparison with authentic samples (see part III in Supporting Materials). The chemical shift zones that have been used for allyl group conversion and yield determination are described in Supporting Materials (see part IV.2). More precisely, in order to facilitate the description of the results, three items were determined for each catalytic test: (i) the allyl group conversion; (ii) the corresponding yields in the different hydrogenated, isomerized, hydroformylated, and hydrohydroxymethylated allyl groups; and (iii) the yield in bis-primary alcohols, with respect to the initial isosorbide moiety. The allyl group conversion and corresponding yields were determined from the ^1^H NMR spectrum, while the yield in bis-primary alcohols was determined by gas chromatography (see parts IV.2 and IV.3 in Supplementary Materials).

In this way, the analysis of the ^1^H NMR spectrum of Figure 1 showed that the preliminary HHM experiment performed with IDE as a substrate gave the following results after 4 h of reaction. The allyl group conversion was equal to 95%. The different related yields were equal to 1% for **(P)** group (Y**_(P)_**(%) = 1), 4% for **(1-P)** group (Y**_(1-P)_**(%) = 4), 16% for aldehyde groups (Y**_(ALD)_**(%) = 16), 4% for hydrogen group (retro-Michael reaction; Y**_(RM)_**(%) = 4), and 70% of primary alcohol groups (Y**_(PA)_**(%) = 70; with a distribution **(4-HB)/(2-HMP)** = 46/54). Furthermore, the analysis of the final reaction medium by gas chromatography indicated a yield in bis-primary alcohols equal to 49%, with respect to the initial isosorbide moiety (Y’**_(BPA)_**(%) = 49).

### 2.3. HHM of IDE: Effect of the TEA Amount and Reaction Time

By following the described experimental conditions, the effect of TEA amount and reaction time was studied (Table 1). An experiment performed without TEA and CO/H_2_ resulted in only isomerization products with a poor allyl group conversion (5%) in 18 h (Table 1, entry 1). The same experiment conducted under CO/H_2_ provided hydrogenated, isomerized, hydroformylated, and retro-Michael products, but no alcohol, after 18 h (Table 1, entry 2).

By increasing the TEA amount from 10 to 200 equivalents with respect to Rh, allyl group conversions quickly reached 100% in 6 h (from a ratio of 20; Table 1, entries 3–7). From a ratio TEA/Rh of 100, the complete conversion of aldehydes to alcohols was observed. Interestingly, the yields in primary alcohols (Y**_(PA)_**) reached 87% (with a linear to branched alcohol ratio around *l*/*b* = 44/56; *l* for **(4-HB)** group, and *b* for **(2-HMP)** group). Logically, good yields in bis-primary alcohols were also obtained (Y’**_(BPA)_** = 67–69%). The regioselectivity related to the primary alcohol group inside of the bis-primary alcohol products was around 17/49/34 for linear-linear(*ll*)/linear-branched(*lb*)/branched-branched(*bb*) ratio, respectively. The products issued from the retro-Michael reaction were always lower than 6%, showing this reaction was minor. Without toluene and with 30 mmol of IDE instead of 10, a total conversion and a yield of 62% in bis-primary alcohols were reached after 18 h (Table 1, entry 8).

The effect of the reaction time was studied at ratio TEA/Rh of 20 and 200 for 4 h and 18 h, to be compared to 6 h (Table 1; entries 9 and 10 to be compared to entry 4 for TEA/Rh = 20, and entries 11 and 12 to be compared to entry 7 for TEA/Rh = 200). For both TEA/Rh ratios, a shorter reaction time (4 h) was not sufficient to reach a total allyl group conversion (see entries 9 and 11). For the TEA/Rh ratio of 20, an increase in reaction time from 6 h to 18 h allowed for a transformation of the residual aldehydes groups present at 6 h (Y**_(ALD)_**(%) = 22, entry 4) into primary alcohol groups, reaching a primary alcohol yield of 89% (Y**_(PA)_**(%) = 89, entry 10; see also the kinetic follow-up of this reaction in Supplementary Materials, part IV.4, Figure S29). For the TEA/Rh ratio of 200, the medium composition stayed logically unchanged between 6 h and 18 h because, at 6 h, all the aldehydes were already completely transformed and the CC double bonds still present at t = 6 h were not reactive, (belonging to **(1-P)** groups; compare entries 7 and 12).

### 2.4. HHM of IDE: Effect of the Nitrogen Ligand

**Scheme 3:**
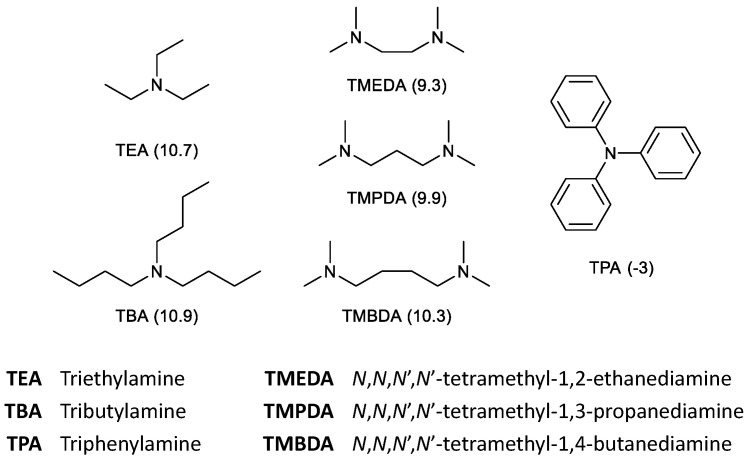
Amines and diamines used (pKa in brackets).

Instead of TEA, other trialkylamines (monodentate TBA and bidentates TMEDA, TMPDA, and TMBDA) and an aromatic amine (TPA) were used as Rh-ligands in the HHM of IDE (Scheme 3). The catalytic result obtained in the presence of TEA as ligand and after 6 h of reaction time was evoked for the comparison (Table 2, entry 1). In the presence of TPA (pKa = −3), no primary alcohol was produced (Table 2, entry 2). This behavior was probably due to the low pKa value. Indeed, some works relative of the HHM of olefins showed that the rhodium/amine complexes were only efficient to produce alcohols for pKa values comprised between 7 and 11 [29]. The other tested amines met this requirement. Consequently, primary alcohols were well produced by using TEA (Y**_(PA)_** = 87%; Table 2 entry 1), TBA (Y**_(PA)_** = 84%; Table 2, entry 3), TMEDA (Y**_(PA)_** = 90%; Table 2, entry 4), TMPDA (Y**_(PA)_** = 89%; Table 2, entry 5), and TMBDA (Y**_(PA)_** = 91%; Table 2, entry 6) at a constant nitrogen/Rh molar ratio of 200 (diamines/Rh ratios were equal to 100).

For these tertiary alkyl-amines and -diamines active in aldehydes hydrogenation, no significant change was observed for the yields in isomerized, saturated, and hydrogen groups (retro-Michael); the yields stayed lower than 10% (entries 1 and 3–6). It is also interesting to notice that the distribution of (*ll*)-, (*lb*)-, and (*bb*)-bis-primary alcohols was not modified in the presence of the bidentate diamines. This behavior was unexpected. Indeed, a modification of the distribution of linear/branched products versus the geometry of the ligand could have been observed. Indeed, in the case of phosphine as a ligand, this phenomenon is widely described in the literature [37], but compared to phosphines, amines are less efficient ligands.

For the diamines, the experiments with a ratio ligand/Rh equal to 200 (nitrogen/Rh of 400) were also performed, but the results were very similar (compare entries 4–6 and 7–9). Nevertheless, the best results in bis-primary alcohol production were obtained with the bidentate diamines (Y’**_(BPA)_** = 74–79%, to be compared to Y’**_(BPA)_** = 69% for TEA).

### 2.5. HHM of IDE: Effect of the Reaction Pressure

As the reaction sequence from alkene to alcohol consumed CO and H_2_ with a ratio of 1:2, experiments with this ratio were performed. In the presence of TEA, similar results were obtained for CO/H_2_ ratios of 1:1 or 1:2 (Table 3, entries 1 and 2). By decreasing the pressure from 80 to 40 bars, the yields in primary alcohol decreased from 87 to 48 (Table 3, entries 2–4). So, a pressure of 80 bars seemed appropriate. The influence of the CO:H_2_ molar ratio was also studied for TMPDA and TMBDA diamines (Table 3, entries 5–8). As for TEA, no modification of the bis-primary alcohol yields was evidenced. In the same way, the variation of the pressure did not impact the distribution of (*ll*)-, (*lb*)-, and (*bb*)-bis-primary alcohols.

## 3. Materials and Methods

All reactions involving metal-amine catalysts were performed under an air atmosphere. The catalytic precursor Rh(acac)(CO)_2_ was purchased from Strem Chemicals and used as received. The different amines were purchased from Acros or Aldrich and used without prior purification. Isosorbide was supplied by Roquette. Syngas (CO:H_2_/1:1) and dihydrogen were provided by the Linde Group in cylinders pressurized at 200 bars. The catalytic experiments were conducted under a fume hood in a room equipped with a CO detector and an explosimeter, both connected to an alarm.

^1^H and ^13^C NMR spectra were recorded at 298 K on a Bruker Avance III HD 300(Wissembourg, France) NanoBay spectrometer equipped with a 5 mm broadband probe BBFO with Z-gradients, operating at 7.05 T field strength (300 MHz for ^1^H nuclei and 75 MHz for ^13^C nuclei). ^1^H and ^13^C chemicals shifts were determined using residual signals of the deuterated solvents and were calibrated vs. SiMe_4_. Assignment of the signals was carried out using 1D (^1^H, ^13^C) and 2D (COSY, HMBC, HMQC) NMR experiments.

Gas chromatography with flame ionization detection (GC-FID) was monitored by analyzing aliquots of the reaction mixture using a Shimadzu GC-2010 Plus apparatus (Noisiel, France) equipped with an RTX-5 capillary column (30 m, 0.25 mm, 0.25 μm). The oven temperature was programmed as follows: initial temperature of 50 °C, increased to 250 °C by 15 °C/min and held for 15 min. The injector and detector temperatures were 250 °C and nitrogen was used as carrier gas at a constant column flow rate of 1.50 mL/min. An aliquot of the sample was injected in split mode.

Products were also analyzed by ESI-MS (electrospray ionization-mass spectrometry) using an AB SCIEX TripleTOF^®^ 5600 mass spectrometer (AB Sciex, Singapore).

Fourier transform infrared spectroscopy (FT-IR) experiments were carried out in the 4000–400 cm^−1^ region with a spectral resolution of 2 cm^−1^ using a Shimadzu IR Prestige-21 spectrometer equipped with a PIKE MIRacle diamond crystal (Noisiel, France).

In a typical catalytic experiment, Rh(acac)(CO)_2_ (12.9 mg, 0.05 mmol, 1 eq.), Triethylamine (1.012 g, 10 mmol, 200 eq.), isosorbide diallyl ether (2.261 g, 10 mmol, 200 eq.), and toluene (6 mL) were added in a 25 mL stainless-steel autoclave (Parr instrument company) equipped with a mechanical stirrer. The reactor was sealed, the reaction mixture was stirred, and the reactor was heated at 80 °C. Then, the reactor was pressurized with 80 bars of CO/H_2_ (1:1). After the appropriate reaction time, the reactor was cooled to room temperature and depressurized. The crude mixture was concentrated to remove the TEA and the solvent. The mixture was then analyzed by ^1^H NMR spectroscopy and GC-FID. All runs were performed at least twice in order to ensure reproducibility.

## 4. Conclusions

The direct functionalization of isosorbide diallyl ether with primary alcohol functions was performed efficiently by the [Rh(acac)(CO)_2_/trialkylamine] catalytic system under CO/H_2_ pressure. The highest yields in bis-primary alcohols were obtained with bidentate diamines, with a maximum value of 79% obtained with TMBDA. The nature of the ligand (monodentate or bidentate), as well as the ligand/Rh and CO/H_2_ ratios, did not modify the linear and branched products distribution. Among these observations, the more surprising result was the absence of different behaviors between monodentate or bidentate amines concerning regioselectivity. Indeed, for all active amines tested, the branched 2-(hydroxymethyl)propyl group systematically formed in a majority compared to the linear 2-hydroxybutyl group with a ratio *l*/*b* of about 45/55. This ratio was rather consistent with a low hindered catalytic species. These results raised questions on the role of amines as a ligand in this reaction, the key factor relative to the amine efficiency being also linked to its pKa value. In this context, some studies are being run to clarify the exact role played by the amine in the catalytic system. Finally, as the introduction of primary alcohol functions allows circumvention of the low overall reactivity of the secondary alcohol groups of isosorbide after a preliminary allylation, the new isosorbide derivatives obtained by this method could be advantageously used to synthesize isosorbide bio-based polyesters and polyurethanes.

## Figures and Tables

**Table 1:** Influence of the TEA amount on IDE hydrohydroxymethylation *^a^*.

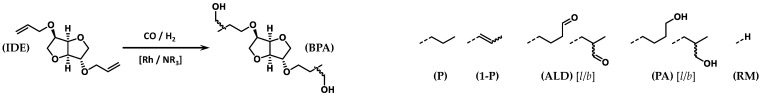									
Entry	TEA/Rh	t (h)	Conv. *^b^* (%)	Y_(P)_ *^c^* (%)	Y_(1-P)_ *^c^* (%)	Y_(ALD)_ *^c^* (%) [*l*/*b*] *^d^*	Y_(PA)_ *^c^* (%) [*l*/*b*] *^e^*	Y_(RM)_ *^c^* (%)	Y’_(BPA)_ *^f^* (%) [*ll/lb/bb*] *^g^*
1*^h^*	0	18	5	0	5	0	0	0	0
2	0	18	100	9	3	82 [50/50]	0	6	0
3	10	6	95	2	12	21 [45/55]	54 [38/62]	6	38 [12/40/48]
4	20	6	100	2	4	22 [45/55]	67 [40/60]	5	44 [12/46/42]
5	50	6	100	2	7	6 [49/51]	80 [45/55]	5	63 [17/49/34]
6	100	6	100	2	5	0	87 [45/55]	6	67 [17/50/33]
7	200	6	100	2	5	0	87 [44/56]	6	69 [17/49/34]
8 *^i^*	1200	18	100	3	6	0	85 [43/57]	6	62 [16/49/35]
9	20	4	79	2	3	38 [50/50]	30 [40/60]	5	22 [12/43/45]
10	20	18	100	3	4	0	89 [46/54]	4	67 [17/50/33]
11	200	4	95	1	4	16 [48/52]	70 [46/54]	4	49 [17/48/35]
12	200	18	100	3	5	0	86 [45/55]	6	67 [17/79/34]

*^a^* Experimental conditions: Rh(acac)(CO)_2_ (12.9 mg, 50 μmol, 1 equiv), IDE (2.26 g, 10 mmol, 200 equiv), TEA (0–1200 equiv), toluene (6 mL), 80 bars of CO/H_2_ (1:1), 80 °C. *^b^* Conv. = allyl groups conversion determined by ^1^H NMR. *^c^* Y**_(X)_** = yield in **(X)** with respect to the initial allyl group, determined by ^1^H NMR; **(P)** = propyl; **(1-P)** = 1-propenyl; **(ALD)** = aldehyde groups = **(2-FP)** + **(3-FP)**; **(PA)** = primary alcohol groups = **(2-HMP)** + **(4-HB)**; **(RM)** = **(2-FP)** grafts that have been cleaved by retro-Michael reaction. *^d^* Linear to branched ratio for aldehydes i.e., **(3-FP)**/**(2-FP)** molar ratio. *^e^* Linear to branched ratio for primary alcohols i.e., **(4-HB)**/**(2-HMP)** molar ratio. *^f^* Y’**_(BPA)_** = yield in bis-primary alcohols with respect to the initial isosorbide moiety, determined by gas chromatography (GC-FID). *^g^* linear-linear/linear-branched/branched-branched ratios for bis-primary alcohols, determined by gas chromatography (GC-FID). *^h^* 1 bar of N_2_ instead of 80 bars of CO/H_2_. *^i^* 30 mmol of IDE instead of 10, and no toluene.

**Table 2:** Influence of the nitrogen compound nature on IDE hydrohydroxymethylation *^a^.*

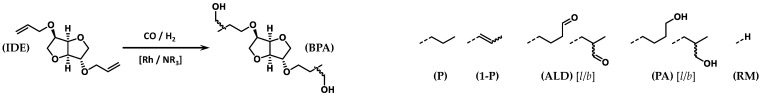								
Entry	Ligand (equiv/Rh)	Conv. *^b^* (%)	Y_(P)_ *^c^* (%)	Y_(1-P)_ *^c^* (%)	Y_(ALD)_ *^c^* (%) [*l*/*b*] *^d^*	Y_(PA)_ *^c^* (%) [*l*/*b*] *^e^*	Y_(RM)_ *^c^* (%)	Y’_(BPA)_ *^f^* (%) [*ll/lb/bb*] *^g^*
1	TEA (200)	100	2	5	0	87 [44/56]	6	69 [17/49/34]
2	TPA (200)	100	9	2	80 [50/50]	0	9	0
3	TBA (200)	100	5	5	0	84 [42/58]	6	60 [16/48/36]
4	TMEDA (100)	100	1	3	0	90 [46/54]	6	74 [16/49/35]
5	TMPDA (100)	100	2	4	0	89 [47/53]	5	75 [15/48/37]
6	TMBDA (100)	100	1	1	0	91 [46/54]	7	79 [16/49/37]
7	TMEDA (200)	100	2	3	0	90 [46/54]	5	75 [15/48/37]
8	TMPDA (200)	100	1	3	0	90 [47/53]	6	76 [16/48/36]
9	TMBDA (200)	100	2	2	0	91 [46/54]	5	79 [16/49/37]

*^a^* Experimental conditions: Rh(acac)(CO)_2_ (12.9 mg, 50 μmol, 1 equiv), IDE (2.26 g, 10 mmol, 200 equiv), Nitrogen compound (10–200 equiv), toluene (6 mL), 80 bars of CO/H_2_ (1:1), 80 °C, 6 h. *^b^* Conv. = allyl groups conversion determined by ^1^H NMR. *^c^* Y**_(X)_** = yield in **(X)** with respect to the initial allyl group, determined by ^1^H NMR; **(P)** = propyl; **(1-P)** = 1-propenyl; **(ALD)** = aldehyde groups = **(2-FP)** + **(3-FP)**; **(PA)** = primary alcohol groups = **(2-HMP)** + **(4-HB)**; **(RM)** = **(2-FP)** grafts that have been cleaved by the retro-Michael reaction. *^d^* Linear to branched ratio for aldehydes i.e., **(3-FP)**/**(2-FP)** molar ratio. *^e^* Linear to branched ratio for primary alcohols i.e., **(4-HB)**/**(2-HMP)** molar ratio. *^f^* Y’**_(BPA)_** = yield in bis-primary alcohols with respect to the initial isosorbide moiety, determined by gas chromatography (GC-FID). *^g^* Linear-linear/linear-branched/branched-branched ratios for bis-primary alcohols, determined by gas chromatography (GC-FID).

**Table 3:** Influence of syngas pressure and composition on IDE hydrohydroxymethylation *^a^.*

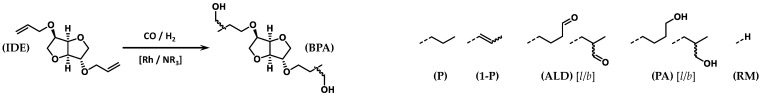									
Entry	Ligand (equiv/Rh)	P (bar) (CO:H_2_)	Conv. *^b^* (%)	Y_(P)_ *^c^* (%)	Y_(1-P)_ *^c^* (%)	Y_(ALD)_ *^c^* (%) [*l*/*b*] *^d^*	Y_(PA)_ *^c^* (%) [*l*/*b*] *^e^*	Y_(RM)_ *^c^* (%)	Y’_(BPA)_ *^f^* (%) [*ll/lb/bb*] *^g^*
1	TEA (200)	80 (1:1)	100	2	5	0	87 [44/56]	6	69 [17/49/34]
2	TEA (200)	80 (1:2)	100	3	4	0	87 [45/55]	6	69 [17/48/35]
3	TEA (200)	60 (1:1)	100	2	13	0	80 [48/52]	5	60 [20/51/29]
4	TEA (200)	40 (1:1)	85	2	18	13 [48/52]	48 [45/55]	4	22 [17/48/35]
5	TMPDA (100)	80 (1:1)	100	2	4	0	89 [47/53]	5	75 [15/48/37]
6	TMPDA (100)	80 (1:2)	100	3	2	0	88 [48/52]	7	74 [16/49/37]
7	TMBDA (100)	80 (1:1)	100	1	1	0	91 [46/54]	7	79 [16/49/37]
8	TMBDA (100)	80 (1:2)	100	3	3	0	87 [46/54]	7	78 [15/49/36]

*^a^* Experimental conditions: Rh(acac)(CO)_2_ (12.9 mg, 50 μmol, 1 equiv), IDE (2.26 g, 10 mmol, 200 equiv), Nitrogen compound (100–200 equiv), toluene (6 mL), CO/H_2_ (1:1 or 1:2; 40–80 bar), 80 °C, 6 h. *^b^* Conv. = allyl groups conversion determined by ^1^H NMR. *^c^* Y**_(X)_** = yield in **(X)** with respect to the initial allyl group, determined by ^1^H NMR; **(P)** = propyl; **(1-P)** = 1-propenyl; **(ALD)** = aldehyde groups = **(2-FP)** + **(3-FP)**; **(PA)** = primary alcohol groups = **(2-HMP)** + **(4-HB)**; **(RM)** = **(2-FP)** grafts that have been cleaved by the retro-Michael reaction. *^d^* Linear to branched ratio for aldehydes i.e., **(3-FP)**/**(2-FP)** molar ratio. *^e^* Linear to branched ratio for primary alcohols i.e., **(4-HB)**/**(2-HMP)** molar ratio. *^f^* Y’**_(BPA)_** = yield in bis-primary alcohols with respect to the initial isosorbide moiety, determined by gas chromatography (GC-FID). *^g^* Linear-linear/linear-branched/branched-branched ratios for bis-primary alcohols, determined by gas chromatography (GC-FID).

## Data Availability

The data presented in this study are available in Supplementary Material.

## Supplementary Materials

The following are available online. The Supplementary Materials contain: a general purpose (part I); an experimental section with the isosorbide diallyl ether synthesis and the general procedure for the reductive hydroformylation catalytic tests (part II); a part dedicated to synthesis and characterization of authentic samples/reaction products, including hydrogenated, isomerized, hydroformylated, and hydrohydroxymethylated isosorbide diallyl ether (part III); and a part explaining the way by which the allyl group conversion, related yields, and regioselectivities were determined by ^1^H NMR analysis; as well as the bis-primary alcohols yield and characteristics by gas chromatography (part IV).

## Sample Availability

Samples of the compounds are available from the authors.

## References

[1] Bozell J.J., Petersen G.R. (2010). Technology development for the production of biobased products from biorefinery carbohydrates—the US Department of Energy’s “Top 10” revisited. Green Chem..

[2] Cole R.T., Kalogeropoulos A.P., Georgiopoulou V.V., Gheorghiade M., Quyyumi A., Yancy C., Butler J. (2011). Hydralazine and Isosorbide Dinitrate in Heart Failure Historical Perspective, Mechanisms, and Future Directions. Circulation.

[3] Delbecq F., Khodadadi M.R., Rodriguez Padron D., Varma R., Len C. (2020). Isosorbide: Recent advances in catalytic production. Mol. Catal..

[4] Tundo P., Aricò F., Gauthier G., Rossi L., Rosamilia A.E., Bevinakatti H.S., Sievert R.L., Newman C.P. (2010). Green Synthesis of Dimethyl Isosorbide. ChemSusChem.

[5] Rose M., Palkovits R. (2012). Isosorbide as a Renewable Platform chemical for Versatile Applications—Quo Vadis?. ChemSusChem.

[6] Feng X., East A.J., Hammond W.B., Zhang Y., Jaffe M. (2011). Overview of advances in sugar-based polymers. Polym. Adv. Technol..

[7] Fenouillot F., Rousseau A., Colomines G., Saint-Loup R., Pascault J.P. (2010). Polymers from renewable 1,4:3,6-dianhydrohexitols (isosorbide, isomannide and isoidide): A review. Prog. Polym. Sci..

[8] Saxon D.J., Luke A.M., Sajjad H., Tolman W.B., Reineke T.M. (2020). Next-generation polymers: Isosorbide as a renewable alternative. Prog. Polym. Sci..

[9] Kricheldorf H.R. (1997). “Sugar Diols” as Building Blocks of Polycondensates. J. Macromol. Sci..

[10] Cognet-Georjon E., Mechin F., Pascault J.P. (1996). New polyurethanes based on 4,4′-diphenylmethane diisocyanate and 1,4:3,6 dianhydrosorbitol, 2. Synthesis and properties of segmented polyurethane elastomers. Macromol. Chem. Phys..

[11] Majdoub M., Loupy A., Fleche G. (1994). Nouveaux polyéthers et polyesters à base d’isosorbide: Synthèse et caractérisation. Eur. Polym. J..

[12] Noordover B.A.J., van Staalduinen V.G., Duchateau R., Koning C.E., van B., Mak M., Heise A., Frissen A.E., van Haveren J. (2006). Co- and Terpolyesters Based on Isosorbide and Succinic Acid for Coating Applications:  Synthesis and Characterization. Biomacromolecules.

[13] Thiyagarajan S., Gootjes L., Vogelzang W., van Haveren J., Lutz M., van Es D.S. (2011). Renewable Rigid Diamines: Efficient, Stereospecific Synthesis of High Purity Isohexide Diamines. ChemSusChem.

[14] Thiyagarajan S., Gootjes L., Vogelzang W., Wu J., van Haveren J., van Es D.S. (2011). Chiral building blocks from biomass: 2,5-diamino-2,5-dideoxy-1,4-3,6-dianhydroiditol. Tetrahedron.

[15] Thiyagarajan S., Wu J., Knoop R., Haveren J., Lutz M., Van Es D. (2014). Isohexide hydroxy esters: Synthesis and application of a new class of biobased AB-type building blocks. Rsc Adv..

[16] Stensrud K. (2013). Monoallyl, Monoglycidyl Ethers and Bisglycidyl Ethers of Isohexides.

[17] Torres G.M., Frauenlob R., Franke R., Börner A. (2015). Production of alcohols via hydroformylation. Catal. Sci. Technol..

[18] Diebolt O., Müller C., Vogt D. (2012). “On-water” rhodium-catalysed hydroformylation for the production of linear alcohols. Catal. Sci. Technol..

[19] Rodrigues F.M.S., Kucmierczyk P.K., Pineiro M., Jackstell R., Franke R., Pereira M.M., Beller M. (2018). Dual Rh−Ru Catalysts for Reductive Hydroformylation of Olefins to Alcohols. ChemSusChem.

[20] Furst M.R.L., Korkmaz V., Gaide T., Seidensticker T., Behr A., Vorholt A.J. (2017). Tandem Reductive Hydroformylation of Castor Oil Derived Substrates and Catalyst Recycling by Selective Product Crystallization. ChemCatChem.

[21] Takahashi K., Yamashita M., Nozaki K. (2012). Tandem Hydroformylation/Hydrogenation of Alkenes to Normal Alcohols Using Rh/Ru Dual Catalyst or Ru Single Component Catalyst. J. Am. Chem. Soc..

[22] Fell B., Shanshool J., Asinger F. (1971). Einstufige oxoalkoholsynthese mit einem Co2(CO)8/Fe(CO)5/N-methylpyrrolidin-katalysatorsystem. J. Organomet. Chem..

[23] Fell B., Geurts A. (1972). Hydroformylierung mit Rhodiumcarbonyl-tert.-Amin-Komplexkatalysatoren. Chem. Ing. Tech..

[24] Cheung L.L.W., Vasapollo G., Alper H. (2012). Synthesis of Alcohols via a Rhodium-Catalyzed Hydroformylation–Reduction Sequence using Tertiary Bidentate Amine Ligands. Adv. Synth. Catal..

[25] Mizoroki T., Kioka M., Suzuki M., Sakatani S., Okumura A., Maruya K.-i. (1984). Behavior of Amine in Rhodium Complex&ndash;Tertiary Amine Catalyst System Active for Hydrogenation of Aldehyde under Oxo Reaction Conditions. Bull. Chem. Soc. Jpn..

[26] Hunter D.L., Moore S.E., Garrou P.E., Dubois R.A. (1985). Dicyclopentadiene hydroformylation catalyzed by RhxCo4-x(CO)12 (x = 4, 2-0)/tertiary amine catalysts. Appl. Catal..

[27] Hunter D.L., Moore S.E., Dubois R.A., Garrou P.E. (1985). Deactivation of rhodium hydroformylation catalysts on amine functionalized organic supports. Appl. Catal..

[28] Alvila L., Pakkanen T.A., Pakkanen T.T., Krause O. (1992). Hydroformylation of olefins catalysed by rhodium and cobalt clusters supported on organic (Dowex) resins. J. Mol. Catal..

[29] Jurewicz A.T., Rollmann L.D., Whitehurst D.D. (1974). Hydroformylation with Rhodium-Amine Complexes. Homogeneous Catalysis—II. Adv. Chem..

[30] Fuchs S., Lichte D., Dittmar M., Meier G., Strutz H., Behr A., Vorholt A.J. (2017). Tertiary Amines as Ligands in a Four-Step Tandem Reaction of Hydroformylation and Hydrogenation: An Alternative Route to Industrial Diol Monomers. ChemCatChem.

[31] Vanbésien T., Monflier E., Hapiot F. (2016). Rhodium-catalyzed one pot synthesis of hydroxymethylated triglycerides. Green Chem..

[32] Rösler T., Ehmann K.R., Köhnke K., Leutzsch M., Wessel N., Vorholt A.J., Leitner W. (2021). Reductive hydroformylation with a selective and highly active rhodium amine system. J. Catal..

[33] Gorbunov D., Nenasheva M., Naranov E., Maximov A., Rosenberg E., Karakhanov E. (2021). Tandem hydroformylation/hydrogenation over novel immobilized Rh-containing catalysts based on tertiary amine-functionalized hybrid inorganic-organic materials. Appl. Catal. A Gen..

[34] Hong J., Radojčić D., Ionescu M., Petrović Z.S., Eastwood R. (2014). Advanced materials from corn: Isosorbide-based epoxy resins. Polym. Chem..

[35] ÇAkmakÇI E., ŞEn F., Kahraman M.V. (2019). Isosorbide Diallyl Based Antibacterial Thiol–Ene Photocured Coatings Containing Polymerizable Fluorous Quaternary Phosphonium Salt. ACS Sustain. Chem. Eng..

[36] Montassier C., Menezo J.C., Moukolo J., Naja J., Barbier J., Boitiaux J.P., Guisnet M., Barrault J., Bouchoule C., Duprez D., Pérot G., Maurel R., Montassier C. (1991). Furanic Derivatives Synthesis from Polyols by Heterogeneous Catalysis Over Metals. Studies in Surface Science and Catalysis.

[37] Jiao Y., Torne M.S., Gracia J., Niemantsverdriet J.W., van Leeuwen P.W.N.M. (2017). Ligand effects in rhodium-catalyzed hydroformylation with bisphosphines: Steric or electronic?. Catal. Sci. Technol..

