# Supplementary material for: Reductive Hydroformylation of Isosorbide Diallyl Ether
Source: Molecules. 2021 Dec 2;26(23):7322. doi: 10.3390/molecules26237322 (PMC8658770; doi:10.3390/molecules26237322)
Supplement: Supplementary file 1 [file molecules-26-07322-s001.zip › molecules-1472185-SI.pdf]

molecules

Supplementary Materials

Reductive Hydroformylation of Isosorbide Diallyl Ether

Jérémy Ternel [1], Adrien Lopes [1,2], Mathieu Sauthier [2], Clothilde Buffe [3], Vincent Wiatz [3], Hervé Bricout [1], Sébastien Tilloy [1,*] and Eric Monflier [1]

[1] Univ. Artois, CNRS, Centrale Lille, Univ. Lille, UMR 8181 – UCCS – Unité de Catalyse et Chimie du Solide, F-62300 Lens, France; jeremy.ternel@univ-artois.fr (J.T.); adrien.figueiredo.lopes@gmail.com (A.L.); herve.bricout@univ-artois.fr (H.B.); eric.monflier@univ-artois.fr (E.M.)
[2] Univ. Lille, CNRS, Centrale Lille, ENSCL, Univ. Artois, UMR 8181, Unité de Catalyse et Chimie du Solide, F-59000 Lille, France; mathieu.sauthier@univ-lille.fr
[3] Roquette Frères, 1 rue de la Haute Loge, 62136 Lestrem, France; clothilde.buffe@roquette.com (C.B.); vincent.wiatz@roquette.com (V.W.)
* Correspondence: sebastien.tilloy@univ-artois.fr (S.T.)

Number of pages:	26
Number of schemes:	1
Number of figures:	29
Number of tables:	1

Table of contents

I) General purpose 3

II) Experimental section 3

II.1 Synthesis and characterization of isosorbide diallyl ether (IDE)........ 3

II.2 General procedure for catalytic experiments........ 6

III) Synthesis and characterization of authentic samples/ reactions products 7

III.1 Hydrogenated IDE (isosorbide dipropyl ether)........ 7

III.2 Isomerized IDE (isosorbide bis(1-propenyl) ether)........ 9

III.3 Hydroformylated IDE (isosorbide bis(2 or 3-formylpropyl) ether)........ 12

III.4 Hydrohydroxymethylated IDE (bis-primary alcohol compound)........ 15

IV) Determination of conversion, yields, and l/b ratios for the catalytic experiments 19

IV.1 Preliminary purpose........ 19

IV.2 Determination of conversion, yields, and l/b ratios from ^{1}H NMR spectra........ 21

IV.3 Determination of yield ($Y'_{(BPA)}$) and of selectivity ratios ($[ll/lb/bb]$) for the bis-primary alcohols from GC-FID chromatograms........ 26

IV.4 Kinetic follow-up of an isosorbide diallyl ether hydrohydroxymethylation catalytic test..... 27

I) General purpose

All reactions involving metal-amine catalysts were performed under an air atmosphere. The Rh precursor was purchased from Strem Chemicals and used as received. The different amines were purchased from Acros or Aldrich and were used without prior purification. Isosorbide was supplied by Roquette. Syngas ($CO:H_2$/1:1) and dihydrogen were provided by the Linde Group in cylinders pressurized at 200 bars. The catalytic experiments were conducted under a fume hood in a room equipped with a CO detector and an explosimeter, both connected to an alarm.

^1H and ^{13}C NMR spectra were recorded at 298 K on a Bruker Avance III HD 300 NanoBay spectrometer equipped with a 5 mm broadband probe BBFO with Z-gradients, operating at 7.05 T field strength (300 MHz for ^1H nuclei and 75 MHz for ^{13}C nuclei). ^1H and ^{13}C chemical shifts were determined using residual signals of the deuterated solvents and were calibrated *vs*. $SiMe_4$. Assignment of the signals was carried out using 1D (^1H, $^{13}C\{^1H\}$, ^{13}C-JMOD) and 2D (COSY, HMBC, HMQC) NMR experiments.

Gas chromatography with flame ionization detection (GC-FID) was monitored by analyzing aliquots of the reaction mixture using a Shimadzu GC-2010 Plus apparatus equipped with an RTX-5 capillary column (30 m, 0.25 mm, and 0.25 μm). The oven temperature was programmed as follows: initial temperature of 50 °C, increased to 250 °C by 15 °C/min and held for 15 min. The injector and detector temperatures were 250 °C, and nitrogen was used as the carrier gas at a constant column flow rate of 1.50 mL/min. An aliquot of the sample was injected in split mode.

The products formed were also analyzed by ESI-MS (electrospray ionization-mass spectrometry) using an AB SCIEX TripleTOF® 5600 mass spectrometer (AB Sciex, Singapore).

Fourier transform infrared spectroscopy (FT-IR) experiments were carried out in the 4000–400 cm^{-1} region with a spectral resolution of 2 cm^{-1} using a Shimadzu IR Prestige-21 spectrometer equipped with a PIKE MIRacle diamond crystal.

II) Experimental section

II.1 Synthesis and characterization of isosorbide diallyl ether (IDE)

Isosorbide (1000 g) is introduced into a 10 L reactor with a cooler and melted at 75 °C. An aqueous solution of NaOH at a 50% weight (3 molar eq. of NaOH, with respect to isosorbide) is then introduced dropwise into the medium. Following this, the reaction medium is maintained at 75 °C for 1 h. Allyl bromide (3 molar eq., with respect to isosorbide) is then added dropwise at 90 °C. After 3 h of addition, the system is maintained at 90 °C for 1 h and the salts that have formed are filtered off. The crude reaction is then treated with ethyl acetate. After decantation and ethyl acetate evaporation, a product—a pale-yellow viscous liquid with 99% yield (1.53 kg)—is obtained. This product

is composed of 92% diallylated isosorbide and 8% monoallylated isosorbide. After three washings with water to remove the monoallylated product, pure diallylated isosorbide is obtained with an 88% yield (1.36 kg).

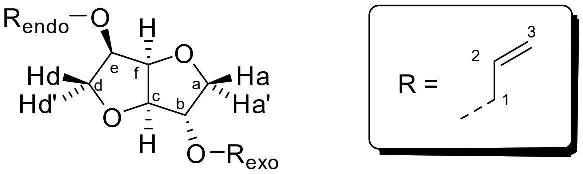

Isosorbide Diallyl Ether (IDE); R = (2-P): colorless liquid. **^{1}H NMR (CDCl$_3$, 300 MHz, 25 °C):** δ 5.95-5.80 (m, 2H, $2H_{2\text{-endo/exo}}$), 5.33-5.11 (m, 4H, $4H_{3\text{-endo/exo}}$), 4.61 (t, *J* = 4.4 Hz, 1H, H_f), 4.49 (d, *J* = 4.3 Hz, 1H, H_c), 4.20 (d, *J* = 4.3 Hz, 1H, $H_{1\text{-endo/exo}}$), 4.10-3.95 (m, 8H, $3H_{1\text{-endo/exo}}$ /H_a/$H_{a'}$/H_b/$H_{d'}$/H_e), 3.57 (t, *J* = 8.1 Hz, 1H, H_d). **^{13}C NMR (CDCl$_3$, 75 MHz, 25 °C):** δ 134.5 ($C_{2\text{-endo/exo}}$), 134.1 ($C_{2\text{-endo/exo}}$), 117.7 ($C_{3\text{-endo/exo}}$), 117.4 ($C_{3\text{-endo/exo}}$), 86.3 (C_c), 83.7 ($C_{b/e}$), 80.2 (C_f), 79.4 ($C_{b/e}$), 73.4 (C_a), 71.6 ($C_{1\text{-endo/exo}}$), 70.5 ($C_{1\text{-endo/exo}}$), 69.8 (C_d) ppm. **ESI-MS:** calculated for $C_{12}H_{18}O_4$ ([M+H$^+$]) = 227.13, found 227.16; ([M+Na$^+$]) = 249.12, found 249.15; ([M+K$^+$]) = 265.12, found 265.12. **FT-IR (cm^{-1}):** 3080, 3020 (ν =CH); 2963, 2934, 2871 (ν CH); 1647 (ν C=C); 1094, 1060 (ν C-O); 995, 921 (γ =CH).

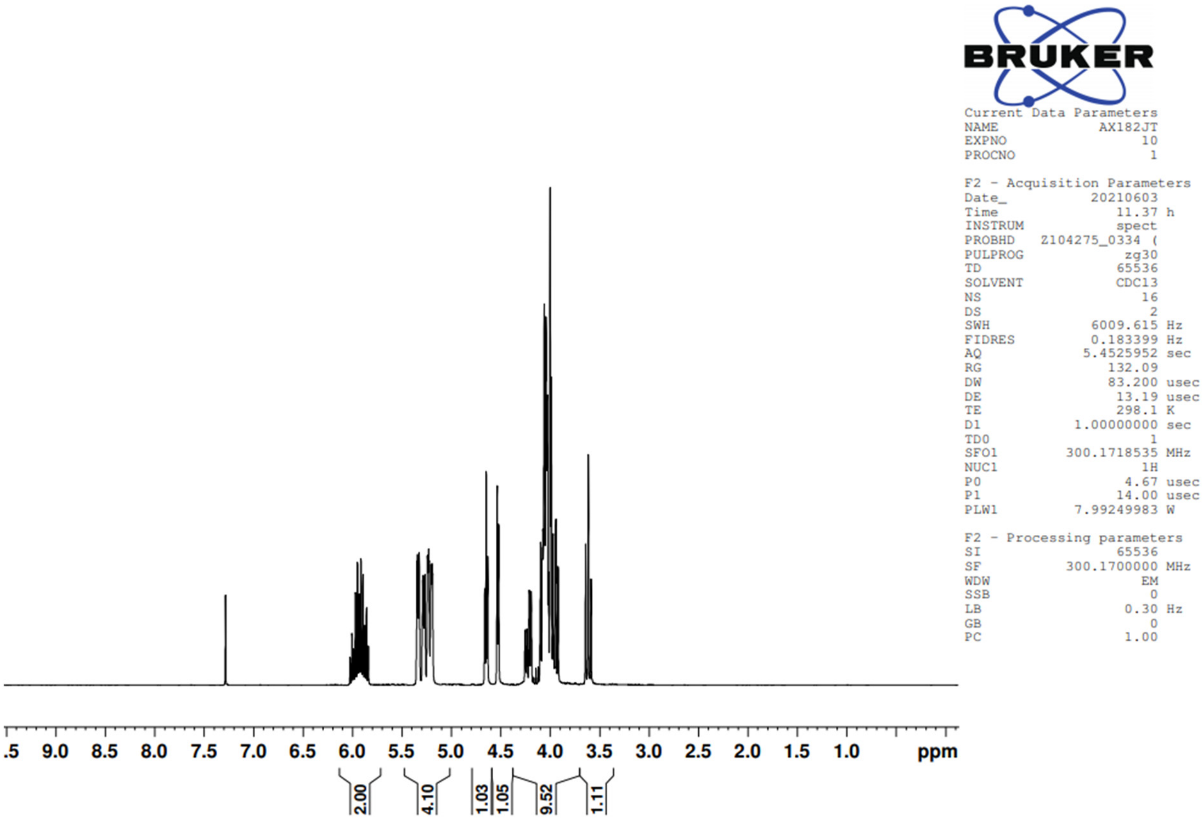

Figure S1. ^{1}H NMR spectrum (300 MHz, CDCl$_3$, 25 °C) of isosorbide diallyl ether; **R = (2-P).**

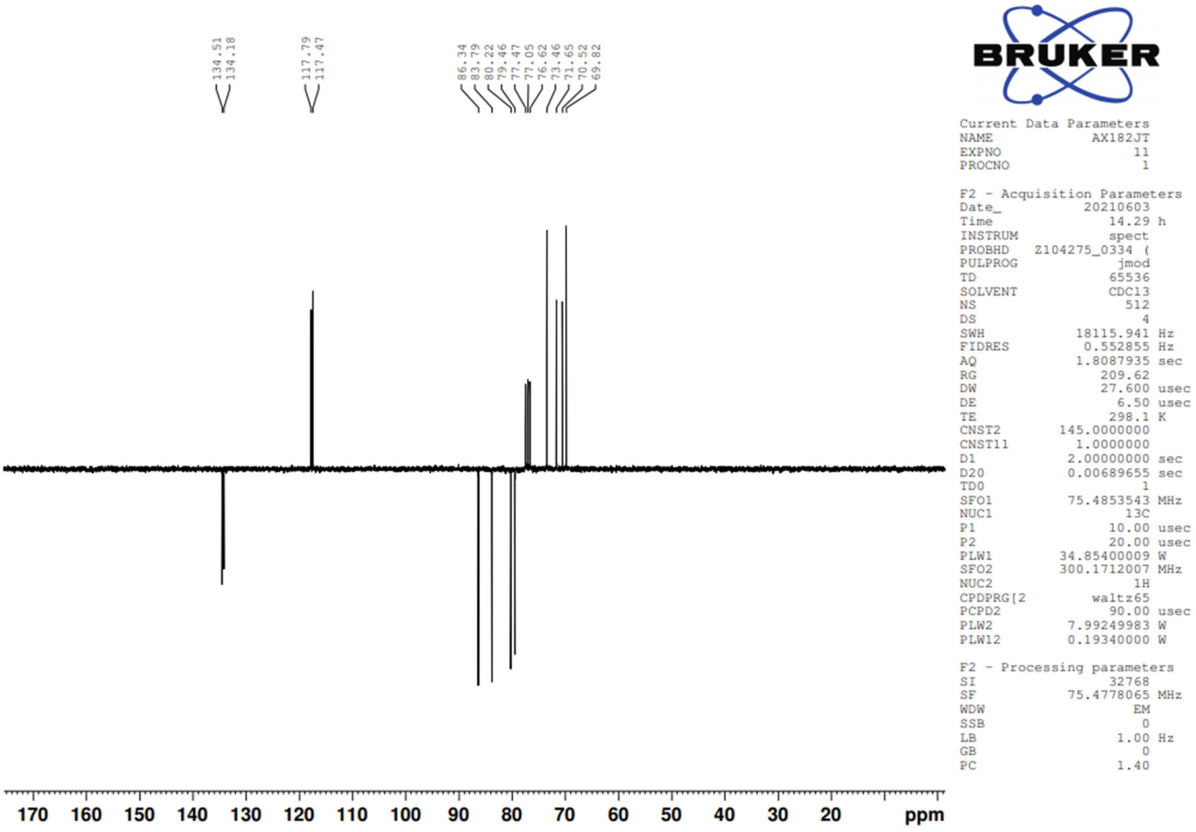

Figure S2. ^{13}C-JMOD NMR spectrum (75 MHz, $CDCl_3$, 25 °C) of isosorbide diallyl ether; **R = (2-P)**

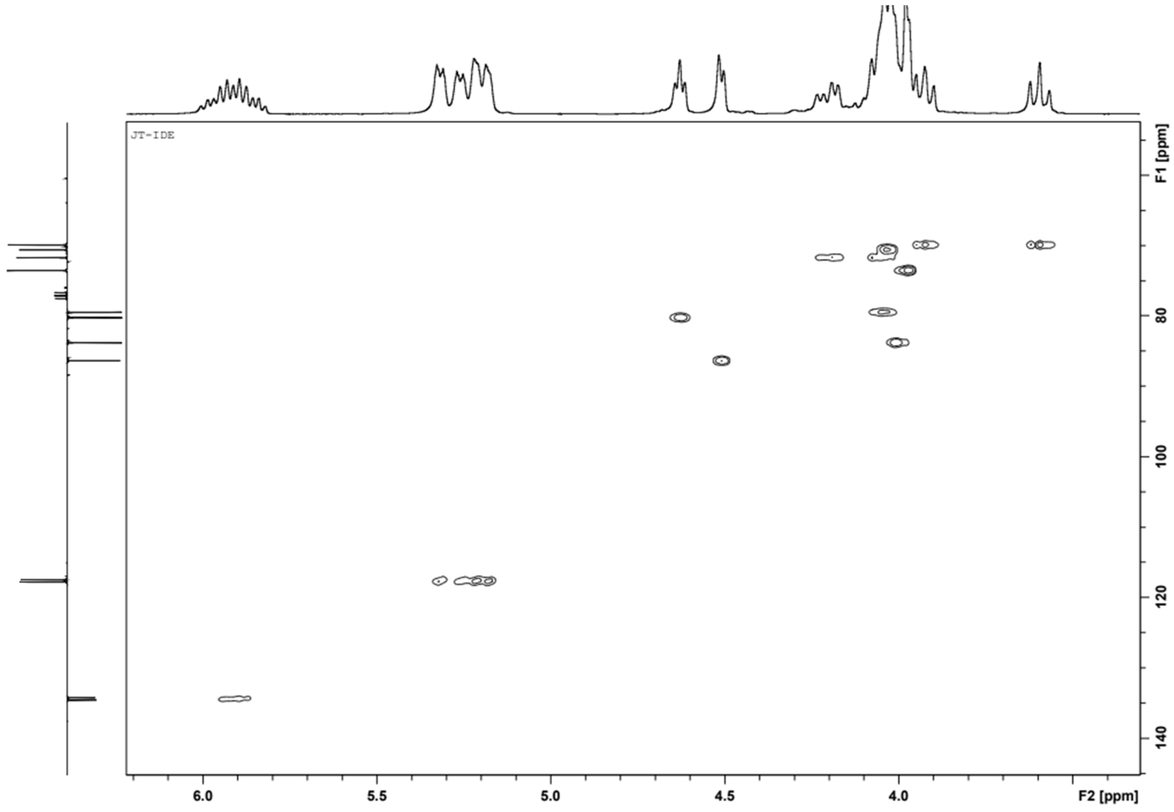

Figure S3. HSQC NMR spectrum (300 MHz, $CDCl_3$, 25 °C) of isosorbide diallyl ether; **R = (2-P).**

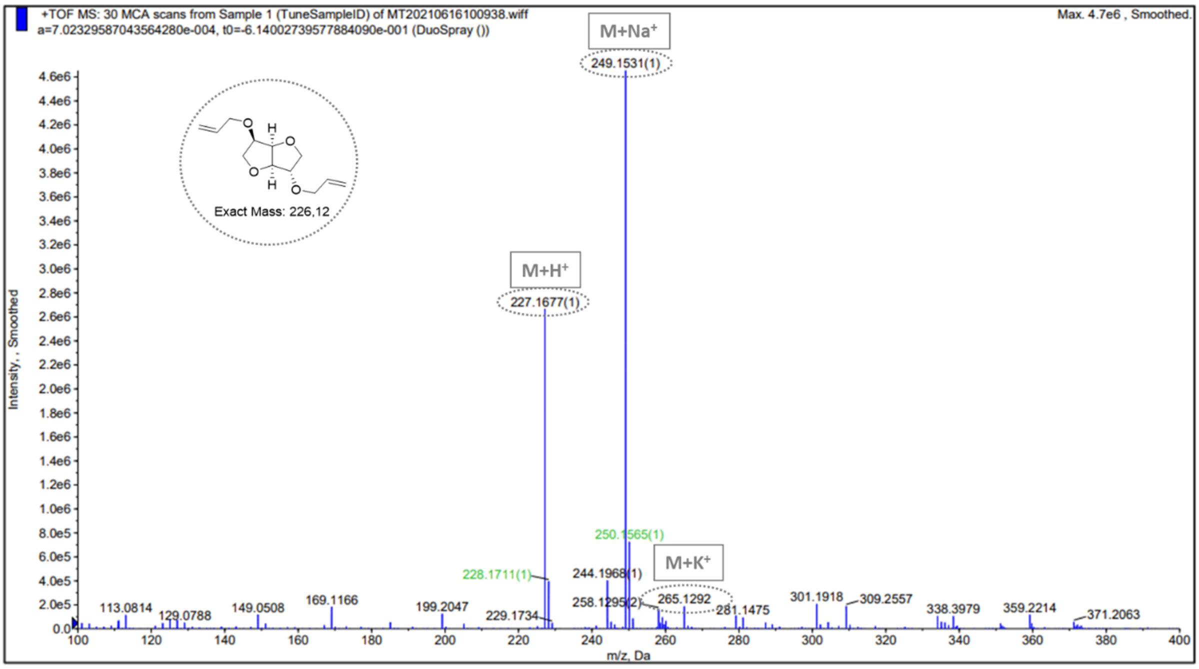

Figure S4. ESI mass spectrum of isosorbide diallyl ether showing only the presence of the diallylated product.

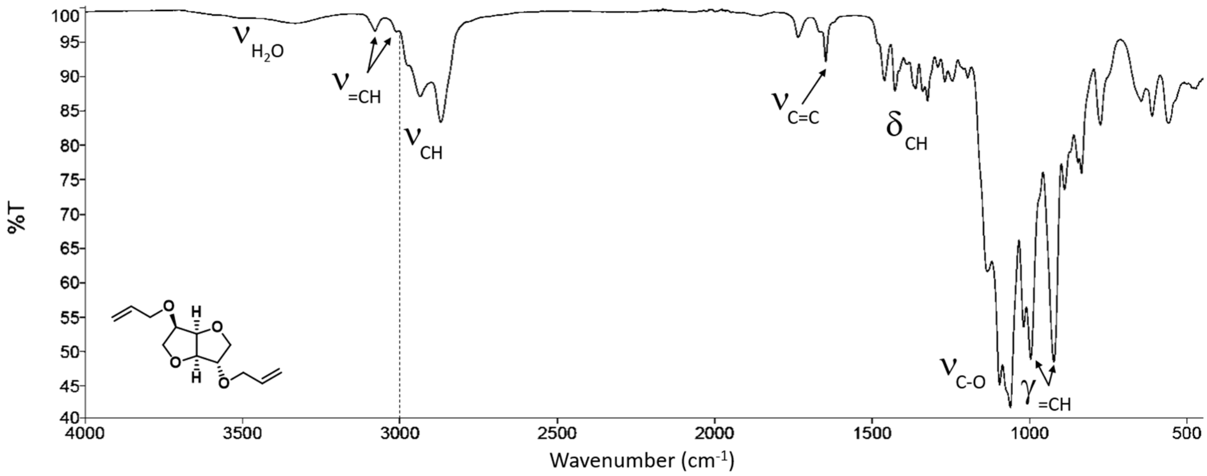

Figure S5. IR spectrum of isosorbide diallyl ether.

II.2 General procedure for catalytic experiments

In a typical catalytic experiment, Rh(acac)$(CO)_2$ (12.9 mg, 0.05 mmol, 1 eq.), NEt_3 (1.012 g, 10 mmol, 200 eq.), isosorbide diallyl ether (2.261 g, 10 mmol, 200 eq.), and toluene (6 mL) were added in a 25 mL stainless-steel autoclave (Parr instrument company) equipped with a mechanical stirrer. The reactor was sealed, the reaction mixture was stirred, and the reactor was heated at 80 °C. Then, the reactor was pressurized with 80 bars of CO/H_2 (1:1). After the appropriate reaction time, the reactor was cooled to room temperature and depressurized. The crude mixture was concentrated to remove the amine and the solvent. Next, the mixture was analyzed by ^{1}H NMR spectroscopy and GC-FID. All runs were performed at least twice in order to ensure reproducibility.

III) Synthesis and characterization of authentic samples/reaction products

III.1 Hydrogenated IDE (isosorbide dipropyl ether)

$[Ir(COD)Cl]_2$ (16.8 mg, 0.025 mmol), NEt_3 (1.012 g, 10 mmol, 200 eq./Ir atom), isosorbide diallyl ether (2.261 g, 10 mmol, 200 eq./Ir atom), and toluene (6 mL) were added under a 25 mL stainless-steel autoclave (Parr instrument company) equipped with a mechanical stirrer. The reactor was sealed, the reaction mixture was stirred, and the reactor was heated at 80 °C. Then, the reactor was pressurized with 80 bars of CO/H_2 (1:1). After 18 h of reaction time, the reactor was cooled to room temperature and depressurized. The crude mixture was concentrated to remove triethylamine and toluene and purified by flash chromatography (ethyl acetate/petroleum ether: from 50/50 to 100/0). Hydrogenated IDE was obtained as a pale-yellow liquid with a 65% isolated yield.

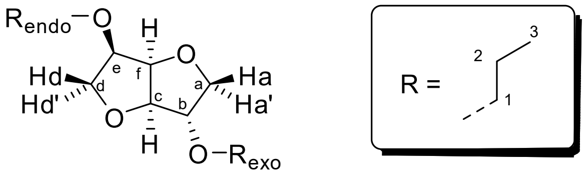

Isosorbide dipropyl ether; **R = (P):** colorless liquid. **^{1}H NMR ($CDCl_3$, 300 MHz, 25 °C)**: δ 4.63 (t, *J* = 4.2 Hz, 1H, H_f), 4.51 (d, *J* = 4.2 Hz, 1H, H_c), 4.0-3.86 (m, 5H, $H_a/H_{a'}/H_b/H_{d'}/H_e$), 3.70-3.32 (m, 5H, $4H_{1\text{-endo/exo}}/H_d$), 1.72-1.52 (m, 4H, $4H_{2\text{-endo/exo}}$), 0.91 (t, *J* = 7.4 Hz, 6H, $6H_{3\text{-endo/exo}}$). **^{13}C NMR ($CDCl_3$, 75 MHz, 25 °C)**: δ 86.4 (C_c), 84.4 ($C_{b/e}$), 80.3 ($C_{b/e}$), 80.1 (C_f), 73.4 (C_a), 72.5 ($C_{1\text{-endo/exo}}$), 71.4 ($C_{1\text{-endo/exo}}$), 69.7 (C_d), 23.0 ($C_{2\text{-endo/exo}}$), 22.9 ($C_{2\text{-endo/exo}}$), 10.50 ($C_{3\text{-endo/exo}}$), 10.40 ($C_{3\text{-endo/exo}}$) ppm. **ESI-MS:** calculated for $C_{12}H_{20}O_4$ ([M+H$^+$]) = 231.16, found 231.19; ([M+Na$^+$]) = 253.15, found 253.18; ([M+K$^+$]) = 269.12, found 269.15. **FT-IR (cm^{-1}):** 2961, 2936, 2875 (ν CH); 1093, 1074 (ν C-O).

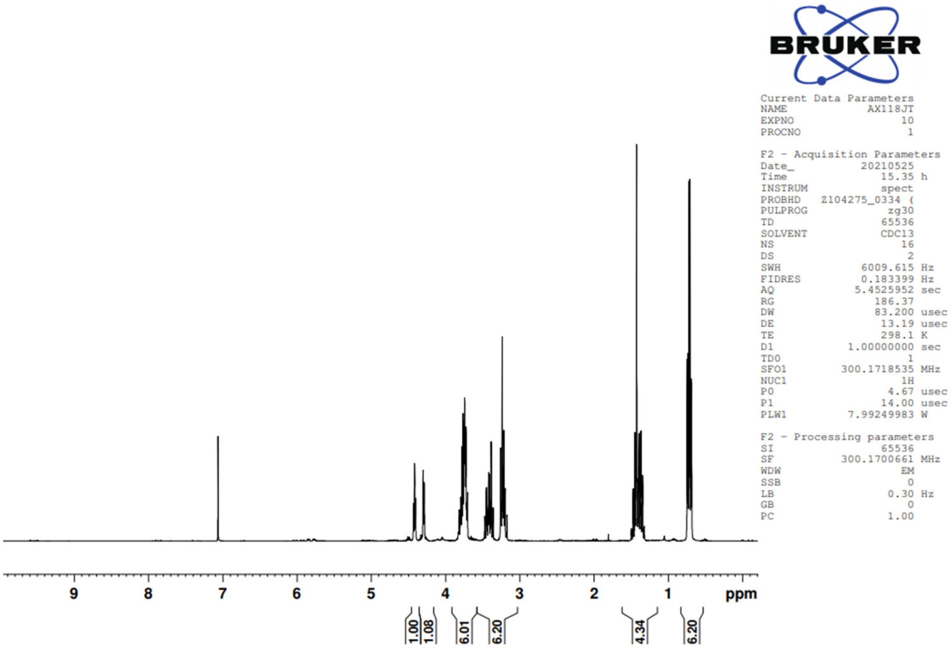

Figure S6. ^{1}H NMR spectrum (300 MHz, $CDCl_3$, 25 °C) of isosorbide dipropyl ether; **R = (P).**

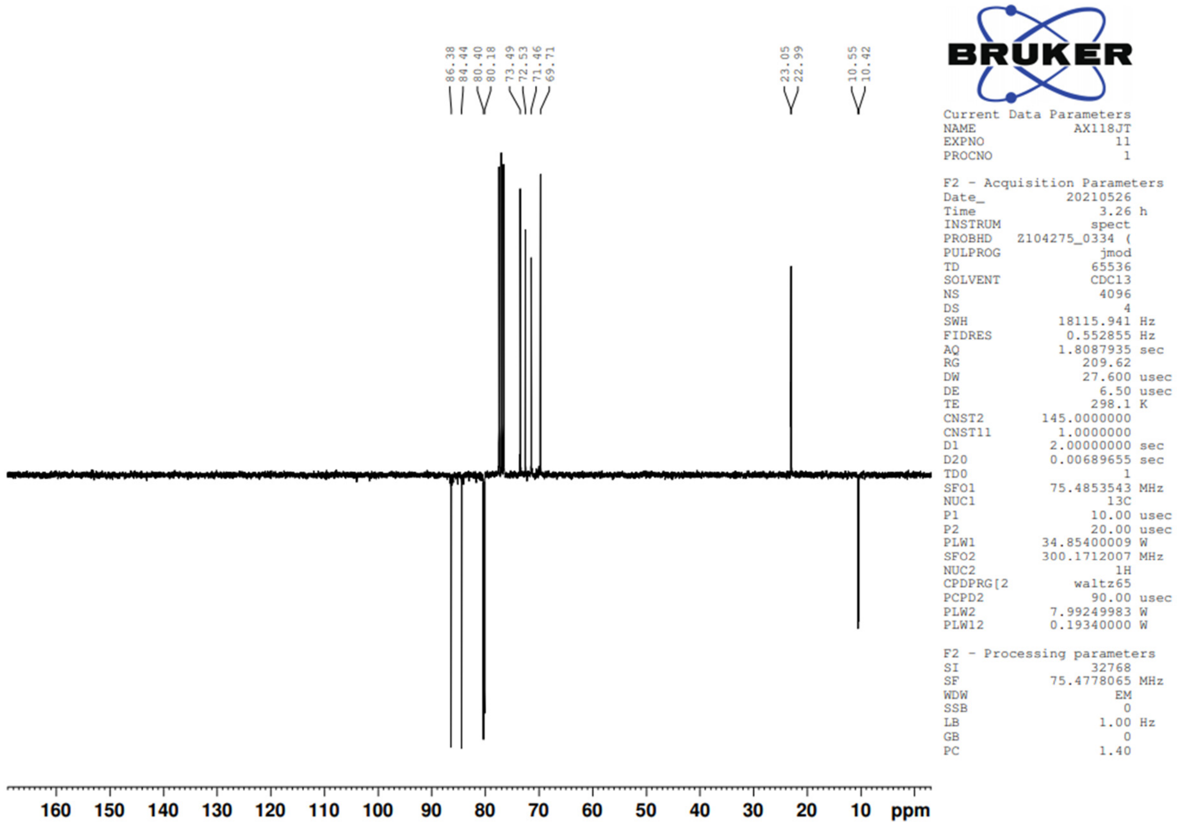

Figure S7. ^{13}C NMR spectrum (75 MHz, $CDCl_3$, 25 °C) of isosorbide dipropyl ether; **R = (P).**

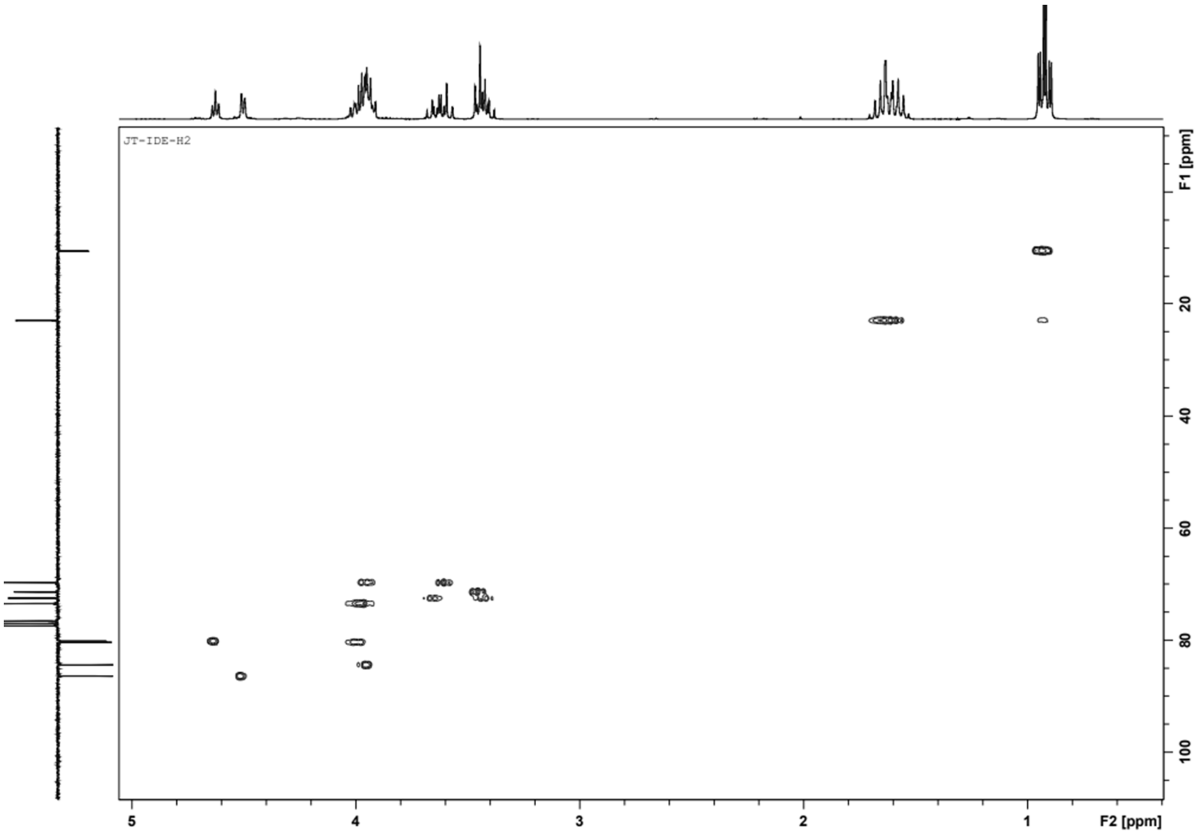

Figure S8. HSQC NMR spectrum (300 MHz, $CDCl_3$, 25 °C) of isosorbide dipropyl ether; **R = (P).**

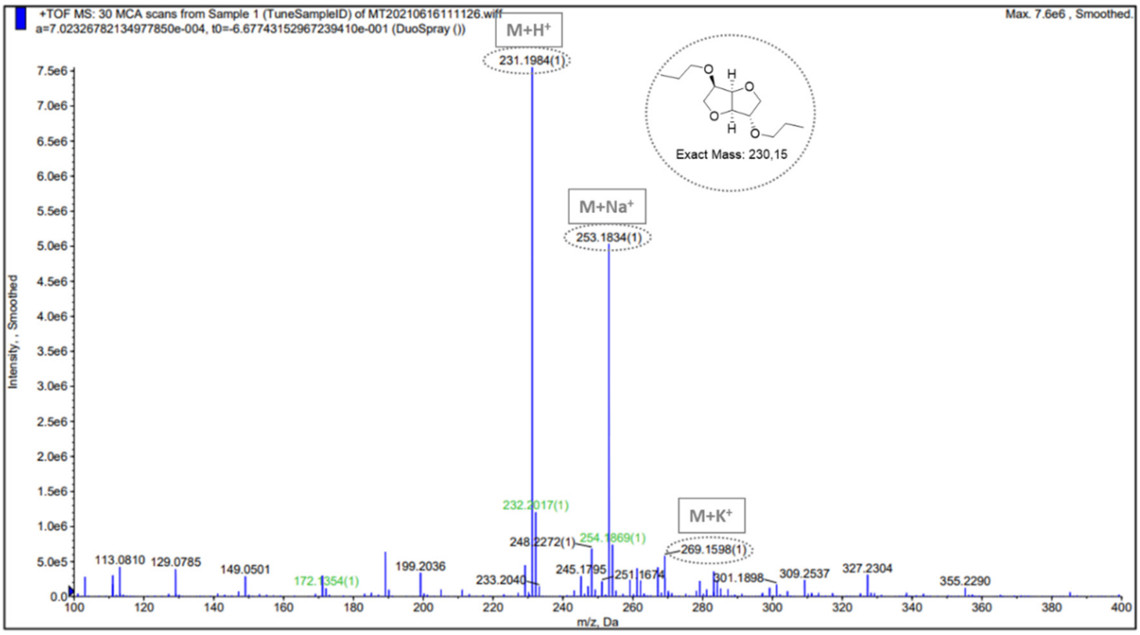

Figure S9. ESI mass spectrum of isosorbide dipropyl ether; **R = (P).**

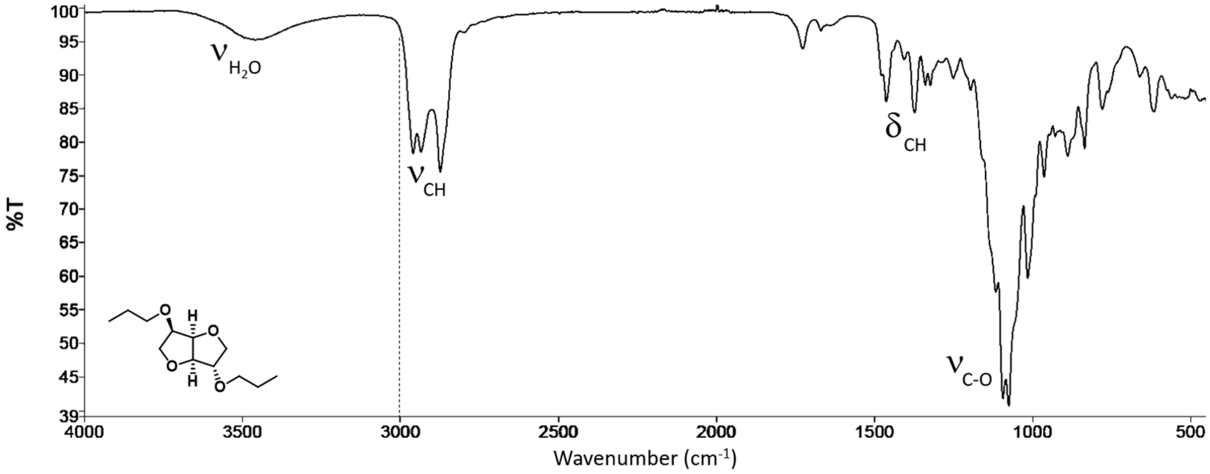

Figure S10. IR spectrum of isosorbide dipropyl ether; **R = (P).**

III.2 Isomerized IDE (isosorbide bis(1-propenyl) ether)

Ru_3CO_{12} (10.6 mg, 0.017 mmol), NEt_3 (1.012 g, 10 mmol, 200 eq./Ru atom), isosorbide diallyl ether (2.261 g, 10 mmol), and toluene (6 mL) were added under a 25 mL stainless-steel autoclave equipped with a mechanical stirrer. The reactor was sealed, the reaction mixture was stirred, and the reactor was heated at 120 °C. Then, the reactor was pressurized with 80 bars of CO/H_2 (1:1). After 6 h of reaction time, the reactor was cooled to room temperature and depressurized. The crude mixture was concentrated to remove NEt_3 and toluene and purified by flash chromatography (ethyl acetate/petroleum ether: from 50/50 to 100/0). Isomerization IDE was obtained as a pale-yellow liquid with a 68% isolated yield.

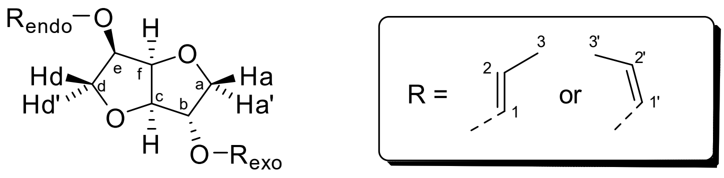

Isosorbide bis(1-propenyl) ether; **R = (1-P):** Obtained as a mixture, colorless liquid. **^{1}H NMR ($CDCl_3$, 300 MHz, 25 °C)**: δ 6.24-5.96 (m, 2H, $H_{1\text{-endo/exo}}$/$H_{1'\text{-endo/exo}}$), 5.04-4.83 (m, 1H, $H_{2\text{-endo/exo}}$), 4.82-4.69 (m, 1H, H_f), 4.61-4.47 (m, 2H, $H_{2'\text{-endo/exo}}$/H_c), 4.39-4.22 (m, 2H, H_b/H_e), 4.11-3.64 (m, 4H, H_a/$H_{a'}$/H_d/$H_{d'}$), 1.42-1.34 (2d, *J* = 1.6 Hz, 6H, 3$H_{3\text{-endo/exo}}$/3$H_{3'\text{-endo/exo}}$). **^{13}C NMR ($CDCl_3$, 75 MHz, 25 °C)**: δ 145.1 (C_1/$C_{1'\text{-endo/exo}}$), 144.2 (C_1/$C_{1'\text{-endo/exo}}$), 144.1 (C_1/$C_{1'\text{-endo/exo}}$), 143.0 (C_1/$C_{1'\text{-endo/exo}}$), 104.2 (C_2/$C_{2'\text{-endo/exo}}$), 102.9 (C_2/$C_{2'\text{-endo/exo}}$), 101.6 (C_2/$C_{2'\text{-endo/exo}}$), 101.3 (C_2/$C_{2'\text{-endo/exo}}$), 86.3 (C_c), 86.0 (C_c), 84.9 ($C_{b/e}$), 84.8 ($C_{b/e}$), 80.7(C_f), 80.6 (C_f), 78.9 ($C_{b/e}$), 78.8 ($C_{b/e}$), 73.6(C_d), 73.4 (C_d), 70.0 (C_a), 69.9 (C_a), 12.5 (C_3/$C_{3'\text{-endo/exo}}$), 12.4 (C_3/$C_{3'\text{-endo/exo}}$), 9.3 (C_3/$C_{3'\text{-endo/exo}}$), 9.2 (C_3/$C_{3'\text{-endo/exo}}$) ppm. **ESI-MS:** calculated for $C_{12}H_{18}O_4$ ([M+H$^+$]) = 227.13, found 227.16; ([M+Na$^+$]) = 249.12, found 249.15; ([M+K$^+$]) = 265.12, found 265.12. **FT-IR (cm^{-1}):** 3043 (ν =CH); 2962, 2932, 2875 (ν CH); 1669 (ν C=C); 1091, 1041 (ν C-O); 927 (γ =CH (*E*)); 728 (γ=CH (*Z*)).

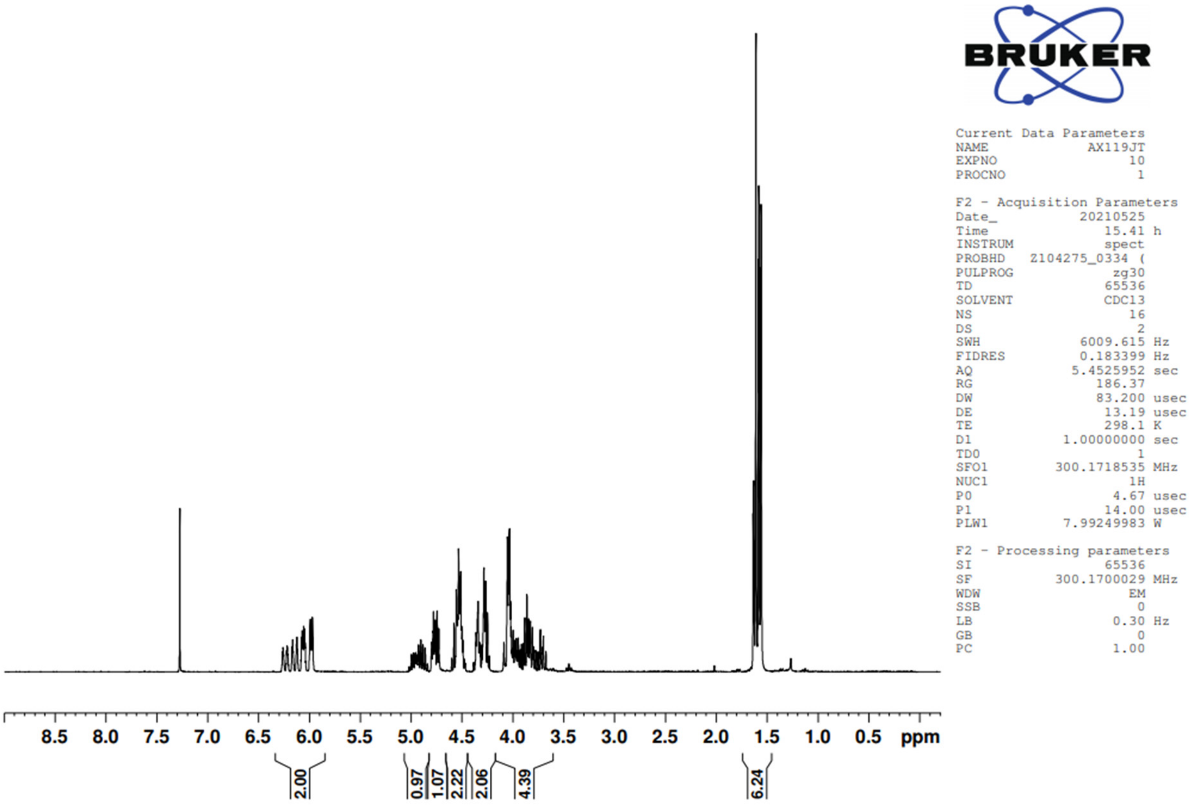

Figure S11. ^{1}H NMR spectrum (300 MHz, $CDCl_3$, 25 °C) of isosorbide bis(1-propenyl) ether; **R = (1-P).**

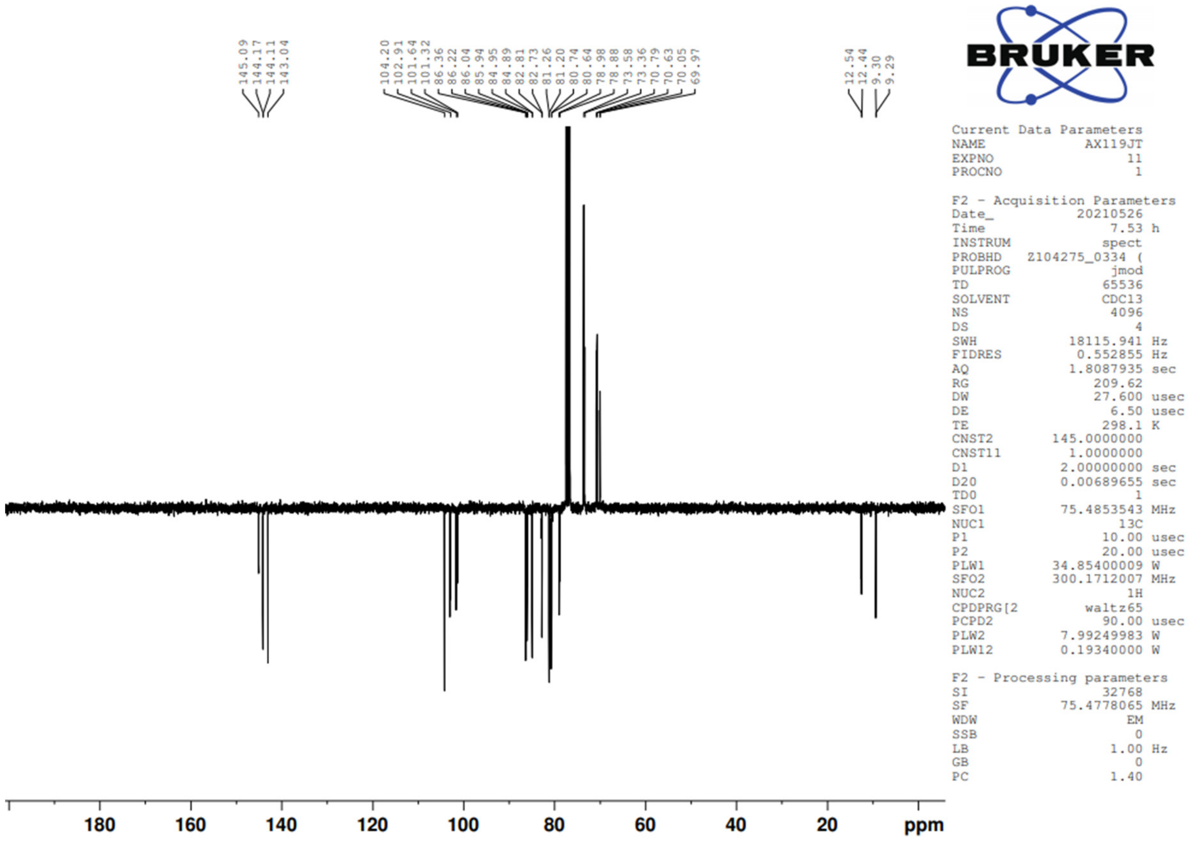

Figure S12. ^{13}C-JMOD NMR spectrum (75 MHz, $CDCl_3$, 25 °C) of isosorbide bis(1-propenyl) ether.

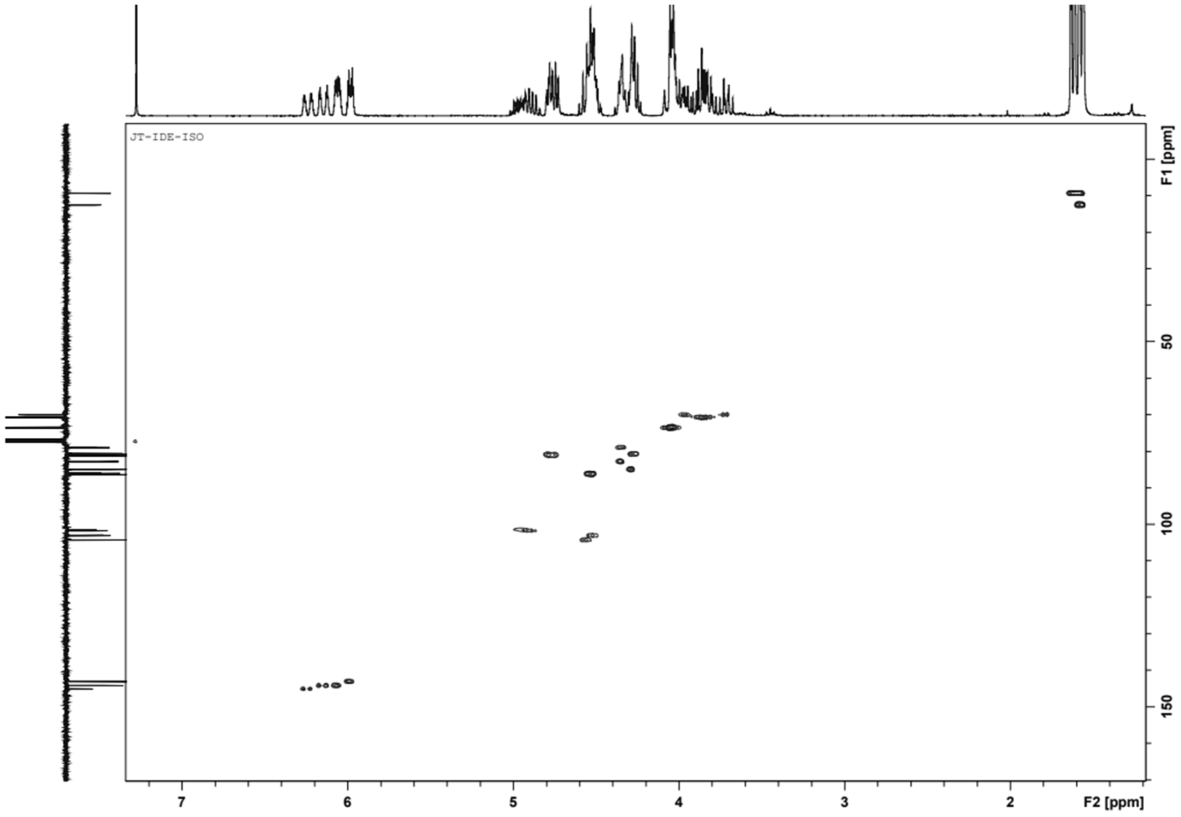

Figure S13. HSQC NMR spectrum (300 MHz, $CDCl_3$, 25 °C) of isosorbide bis(1-propenyl) ether; **R = (1-P).**

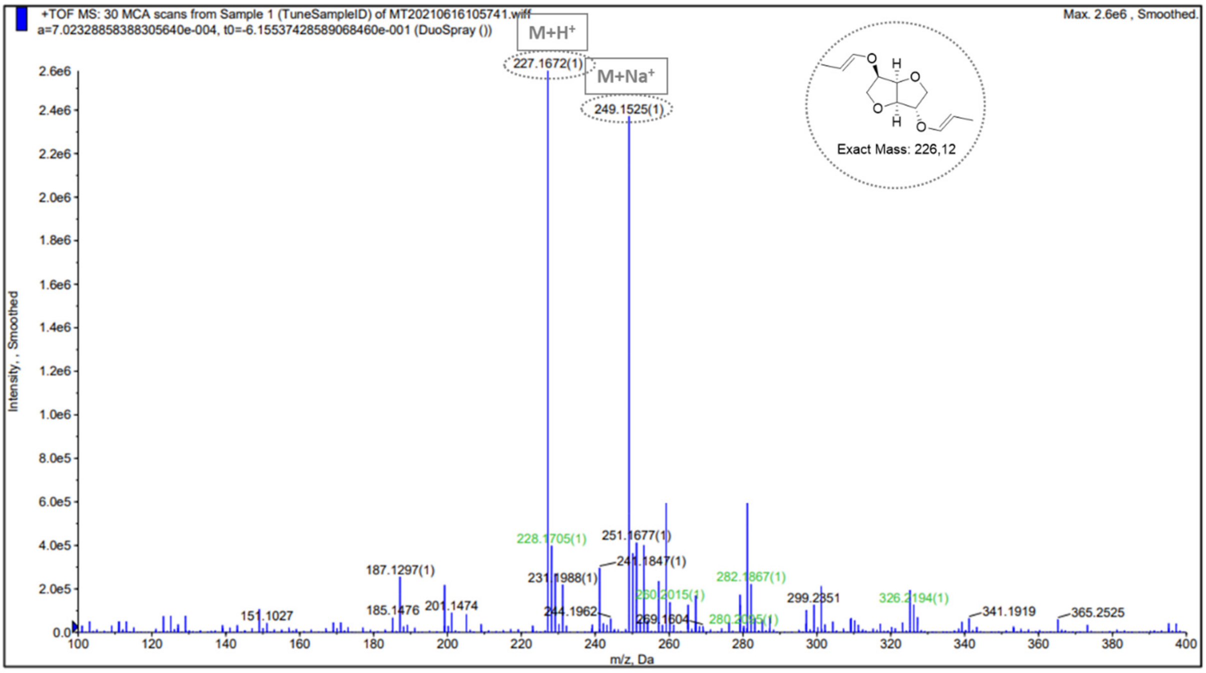

Figure S14. ESI mass spectrum of isosorbide bis(1-propenyl) ether; **R = (1-P).**

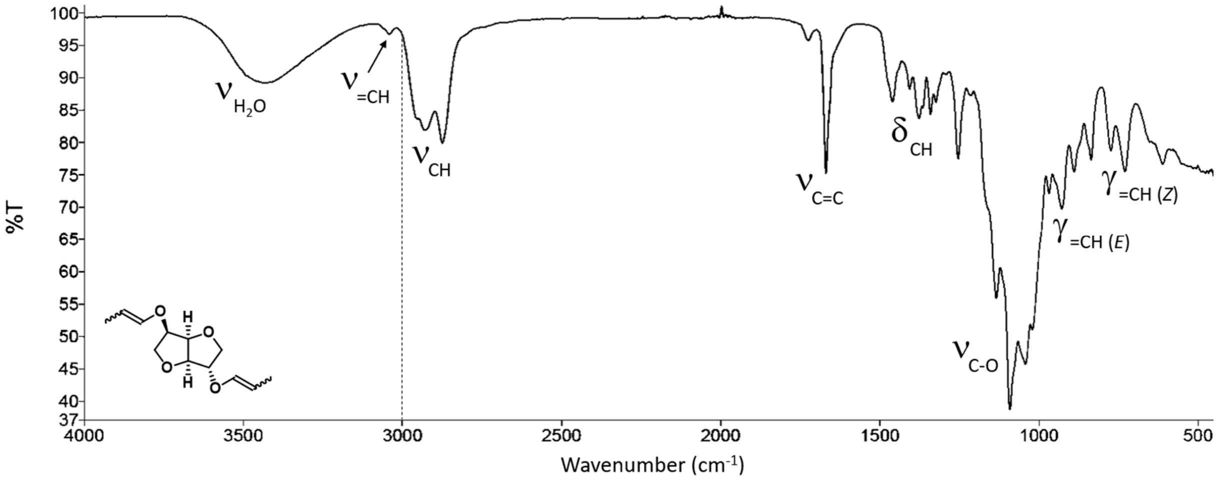

Figure S15. IR spectrum of isosorbide bis(1-propenyl) ether; **R = (1-P).**

III.3 Hydroformylated IDE (isosorbide bis(2 or 3-formylpropyl) ether)

Rh(acac)(CO)$_2$ (12.9 mg, 0.05 mmol, 1 eq.), isosorbide diallyl ether (2.261 g, 10 mmol, 200 eq.), and toluene (6 mL) were added to a 25 mL stainless-steel autoclave (Parr instrument company) equipped with a mechanical stirrer. The reactor was sealed, the reaction mixture was stirred, and the reactor was heated at 80 °C. Then, the reactor was pressurized with 80 bars of CO/H_2 (1:1). After 18 h of reaction time, the reactor was cooled to room temperature and depressurized. The crude mixture was concentrated to remove the amine and the solvent. The mixture was then purified by flash chromatography (ethyl acetate/petroleum ether/methanol: from 50/50/0 to 100/0/0 to 80/0/20). Hydroformylated IDE was obtained as a yellow liquid in a 60% isolated yield.

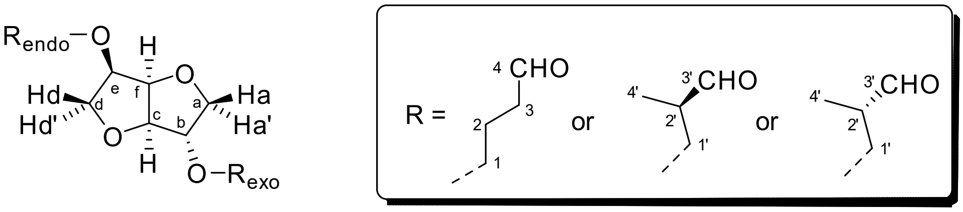

Hydroformylated IDE; **R = (2-FP) or (3-FP):** Obtained as a mixture with a ratio l/b of 50/50; colorless liquid. **^{1}H NMR (CDCl$_3$, 300 MHz, 25 °C)** : δ 9.70 (t, *J* = 1.69 *Hz*, 0.5H, $H_{4\text{-endo/exo}}$), 9.68 (t, *J* = 1.69 *Hz*, 0.5H, $H_{4\text{-endo/exo}}$), 9.64-9.59 (m, 1H, $H_{3'\text{-endo/exo/R/S}}$), 4.64 (t, *J* = 4.2 Hz, 0.25H, H_f), 4.59 (t, *J* = 4.2 Hz, 0.25H, H_f), 4.49 (d, *J* = 4.2 Hz, 0.25H, H_c), 4.44 (d, *J* = 4.2 Hz, 0.25H, H_c), 4.13-3.79 (m, 5H, $H_a/H_{a'}/H_b/H_{d'}/H_e$), 3.73-3.37 (m, 5H, $H_d/2H_{1'\text{-endo/exo}}/2H_{1\text{-endo/exo}}$), 2.75-2.58 (m, *J* = 6.98 *Hz* , 1H, $H_{2'\text{-endo/exo}}$), 2.52 (t, *J* = 6.98 *Hz* , 2H, $2H_{3\text{-endo/exo}}$), 1.90-1.80 (m, 2H, $2H_{2\text{-endo/exo}}$) 1.06-1.04 (2d, *J* = 7.15 *Hz* , 3H, $H_{4'\text{-endo/exo}}/H_{4'\text{-endo/exo}}$) ppm. **^{13}C NMR (CDCl$_3$, 75 MHz, 25 °C)**: δ 203.3 ($C_{4\text{-endo/exo}}$), 203.6 ($C_{4\text{- endo/exo}}$), 202.2 ($C_{3'\text{- endo/exo}}$), 201.9 ($C_{3'\text{- endo/exo}}$), 86.2 (C_c), 86.1 (C_c), 84.6 (C_b), 84.4(C_b), 80.6 (C_e), 80.3 (C_e), 80.2 (C_f), 80.1 (C_f), 73.1 (C_a), 73.0 (C_a), 70.4 (C_d), 69.9 (C_d), 69.4 ($C_{1'\text{-endo/exo}}$), 69.3 ($C_{1\text{-endo/exo}}$), 68.4 ($C_{1'\text{-endo/exo}}$), 68.3 ($C_{1\text{-endo/exo}}$), 46.8 ($C_{2'\text{-endo/exo}}$), 46.6 ($C_{2'\text{-endo/exo}}$), 40.8 ($C_{3\text{-endo/exo}}$), 40.7 ($C_{3\text{-endo/exo}}$), 22.5 ($C_{2\text{-endo/exo}}$), 22.4 ($C_{2\text{-endo/exo}}$), 10.6 ($C_{4'\text{-endo/exo}}$), 10.5 ($C_{4'\text{-endo/exo}}$) ppm. **ESI-MS:** calculated for $C_{14}H_{22}O_6$ ([M+H$^+$]) = 287.15, found 287.19. **FT-IR (cm^{-1}):** 2936, 2875 (ν CH); 2810, 2729 (ν (O=)CH, Fermi resonance); 1719 (ν C=O); 1086 (ν C-O).

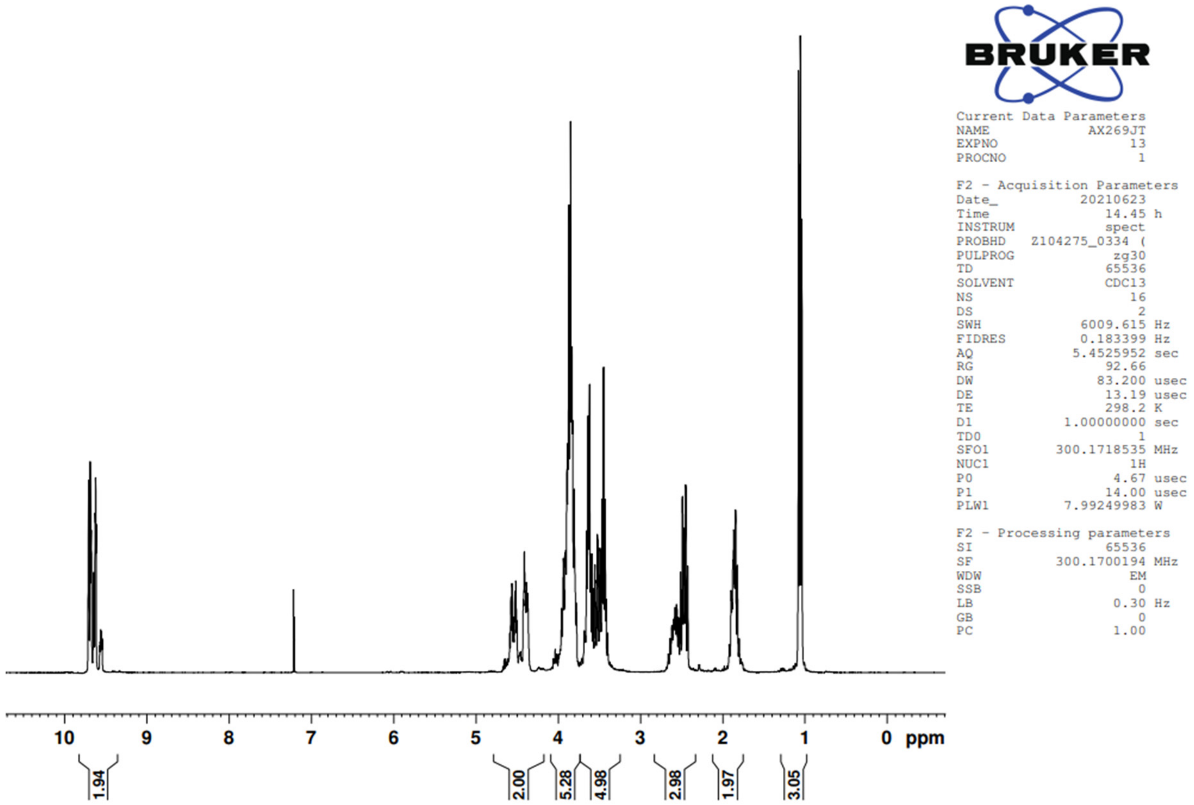

Figure S16. ^{1}H NMR spectrum (300 MHz, CDCl$_3$, 25 °C) of hydroformylated IDE; **R = (2-FP) or (3-FP).**

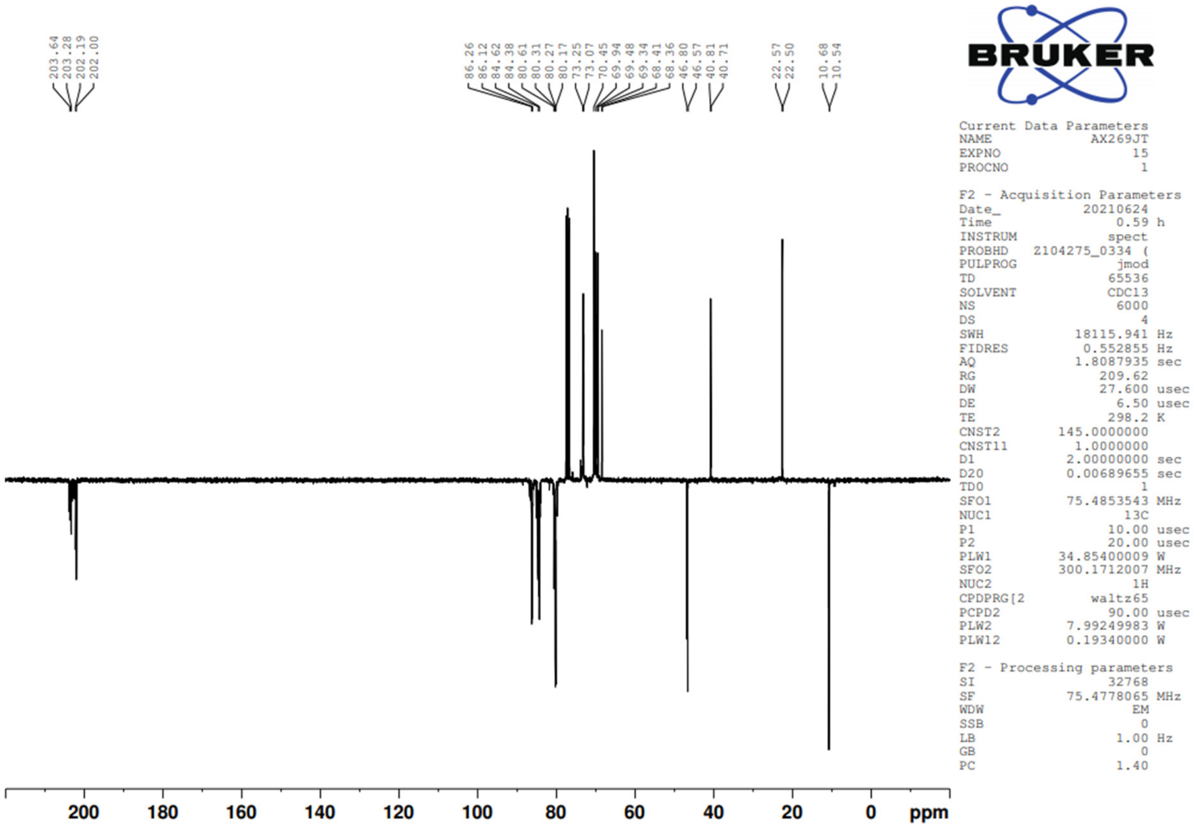

Figure S17. ^{13}C-JMOD NMR spectrum (75 MHz, $CDCl_3$, 25 °C) of hydroformylated IDE; **R = (2-FP) or (3-FP).**

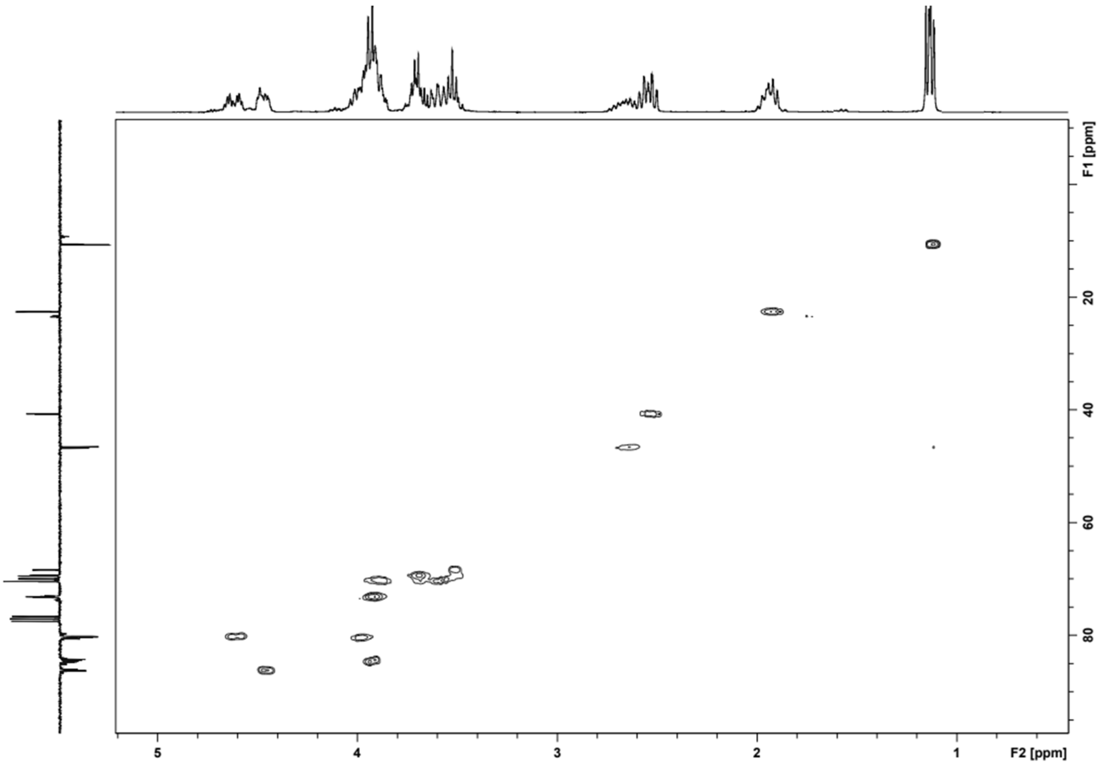

Figure S18. HSQC NMR spectrum (300 MHz, $CDCl_3$, 25 °C) of hydroformylated IDE; **R = (2-FP) or (3-FP).**

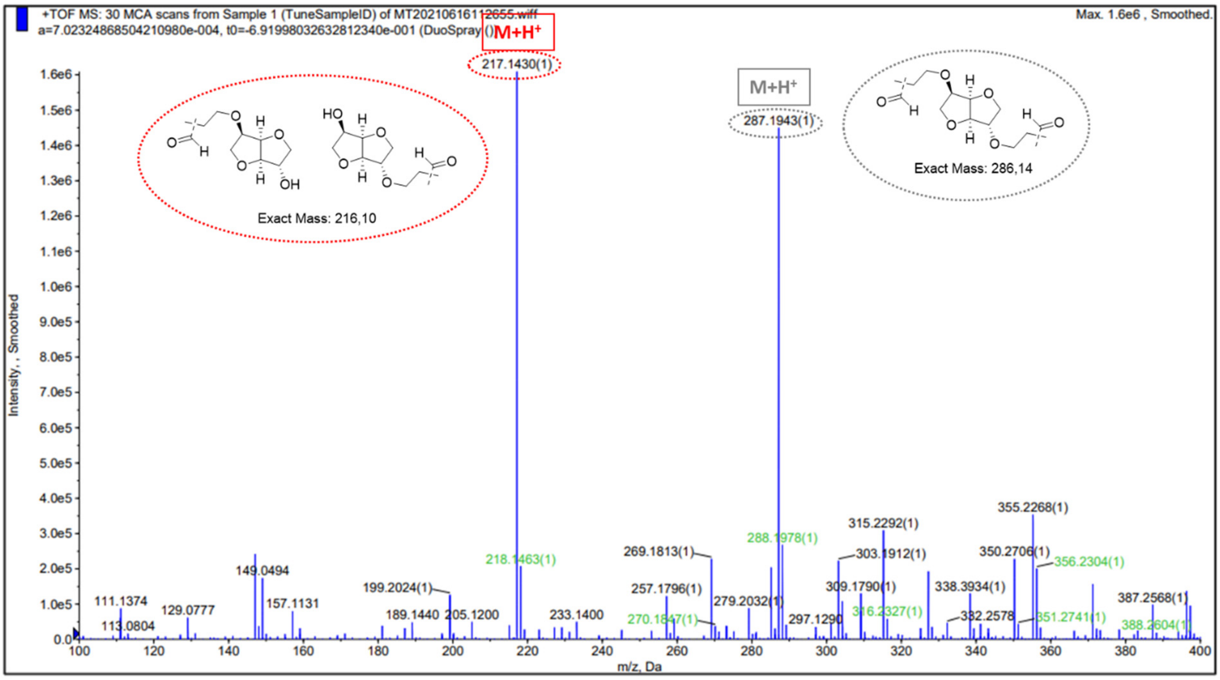

Figure S19. ESI mass spectrum of hydroformylated IDE showing the presence of mono- and bis- aldehyde products; **R = (2-FP) or (3-FP).**

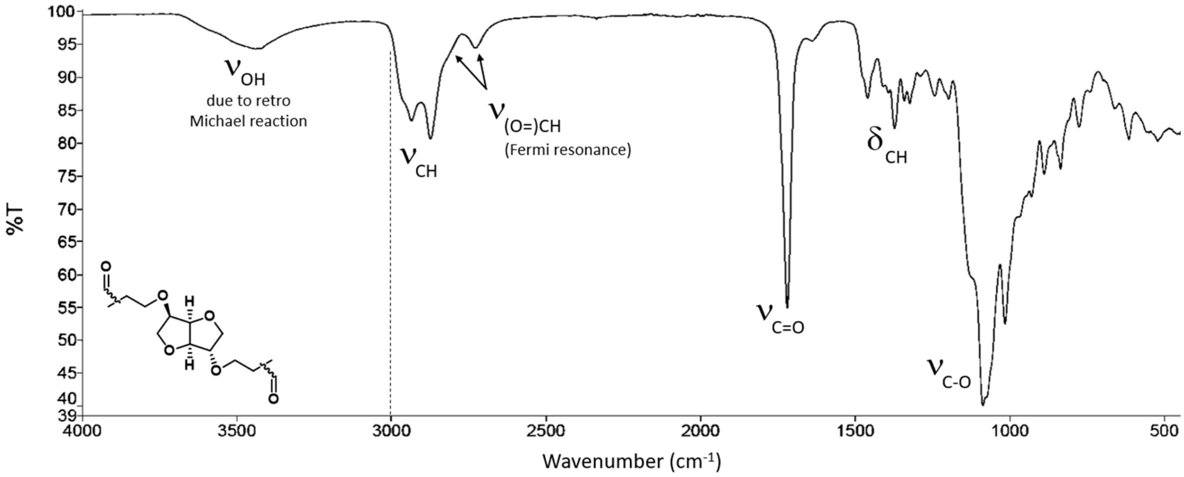

Figure S20. IR spectrum of hydroformylated IDE.

III.4 Hydrohydroxymethylated IDE (bis-primary alcohol compound)

Rh(acac)$(CO)_2$ (12.9 mg, 0.05 mmol, 1 eq.), NEt_3 (1.012 g, 10 mmol, 200 eq.), isosorbide diallyl ether (2.261 g, 10 mmol, 200 eq.), and toluene (6 mL) were added in a 25 mL stainless-steel autoclave (Parr instrument company) equipped with a mechanical stirrer. The reactor was sealed, the reaction mixture was stirred, and the reactor was heated at 80 °C. Then, the reactor was pressurized with 80 bars of CO/H_2 (1:1). After 6 h of reaction time, the reactor was cooled to room temperature and depressurized. The crude mixture was concentrated to remove the amine and the solvent and then purified by flash chromatography (ethyl acetate/petroleum ether/methanol: from 50/50/0 to 100/0/0, and then to 80/0/20). Hydrohydroxymethylated IDE was obtained as a yellow liquid with a 45% isolated yield.

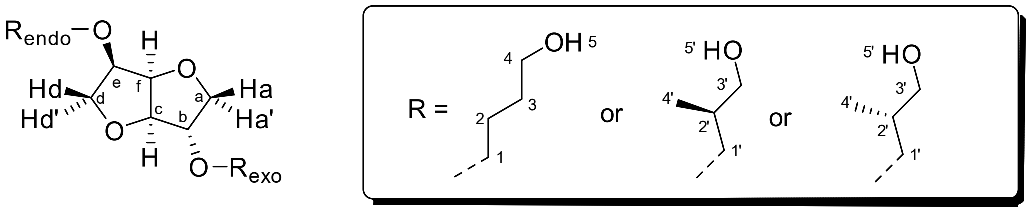

Hydrohydroxymethylated IDE; R = (4-HB) or (2-HMP): Obtained as an inseparable mixture with a ratio l/b of 50/50; colorless liquid. **^{1}H NMR (CDCl$_3$, 300 MHz, 25 °C)**: 4.56 (t, *J* = 4.3 Hz, 1H, H$_f$), 4.43 (d, *J* = 4.2 Hz, 1H, H$_c$), 3.96-3.76 (m, 5H, H$_a$/H$_{a'}$/H$_b$/H$_{d'}$/H$_e$), 3.73-3.27 (m, 9H, H$_d$/H$_{1\text{-endo/exo}}$ /H$_{1'\text{-endo/exo}}$ /H$_{3'\text{-endo/exo}}$ /H$_{4\text{-endo/exo}}$), 2.95 (S$_{br}$, 2H, H$_{5\text{-endo/exo}}$/H$_{5'\text{-endo/exo}}$), 1.95-1.82 (m, 1H, H$_{2'\text{-endo/exo}}$), 1.71-1.44 (m, 4H, 2H$_{2\text{-endo/exo}}$ /2H$_{3\text{-endo/exo}}$), 0.84-0.80 (d, *J* = 7.10 Hz, 3H, 3H$_{4'\text{-endo/exo}}$) ppm. **^{13}C NMR (CDCl$_3$, 75 MHz, 25 °C)**: 86.1 (C$_c$), 86.0 (C$_c$), 84.3 (C$_{b/e}$), 84.2 (C$_{b/e}$), 80.2(C$_b$/$_e$), 80.1 (C$_b$/$_e$), 79.3 (C$_f$), 79.1 (C$_f$), 73.2 (C$_a$), 73.1 (C$_a$), 72.9 (C$_{1'}$), 72.5 (C$_{1'}$), 70.6 (C$_1$), 70.5 (C$_1$), 69.9 (C$_d$), 69.8 (C$_d$), 66.5 (C$_{3'}$), 66.4 (C$_{3'}$), 62.3 (C$_4$), 62.2 (C$_4$), 34.8 (C$_{2'\text{-endo/exo}}$), 34.6 (C$_{2'\text{-endo/exo}}$), 28.7 (C$_{2\text{-endo/exo}}$ /C$_{3\text{-endo/exo}}$), 28.6 (C$_{2\text{-endo/exo}}$ /C$_{3\text{-endo/exo}}$), 26.5 (C$_{2\text{-endo/exo}}$ /C$_{3\text{-endo/exo}}$), 26.2 (C$_{2\text{-endo/exo}}$ /C$_{3\text{-endo/exo}}$), 13.4 (C$_{4'\text{-endo/exo}}$), 9.6 (C$_{4'\text{-endo/exo}}$) ppm. **ESI-MS:** calculated for $C_{14}H_{26}O_6$ ([M+H$^+$]) = 291.18, found 291.23; ([M+Na$^+$]) = 313.15, found 313.21; ([M+K$^+$]) = 329.15, found 329.19. **FT-IR (cm^{-1}):** 3392 (ν OH); 2938, 2873 (ν CH); 1054 (ν C-O).

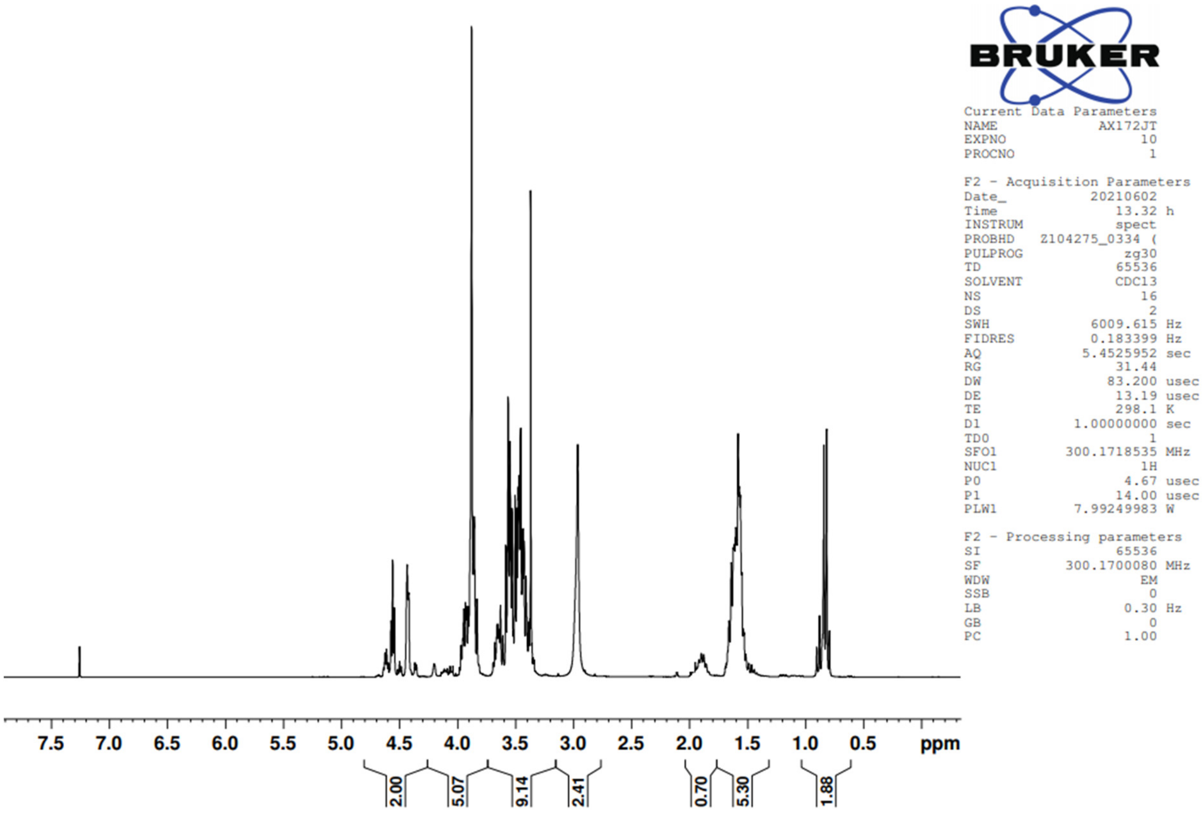

Figure S21. ^{1}H NMR spectrum (300 MHz, CDCl$_3$, 25 °C) of hydrohydroxymethylated IDE; **R = (4-HB) or (2-HMP).**

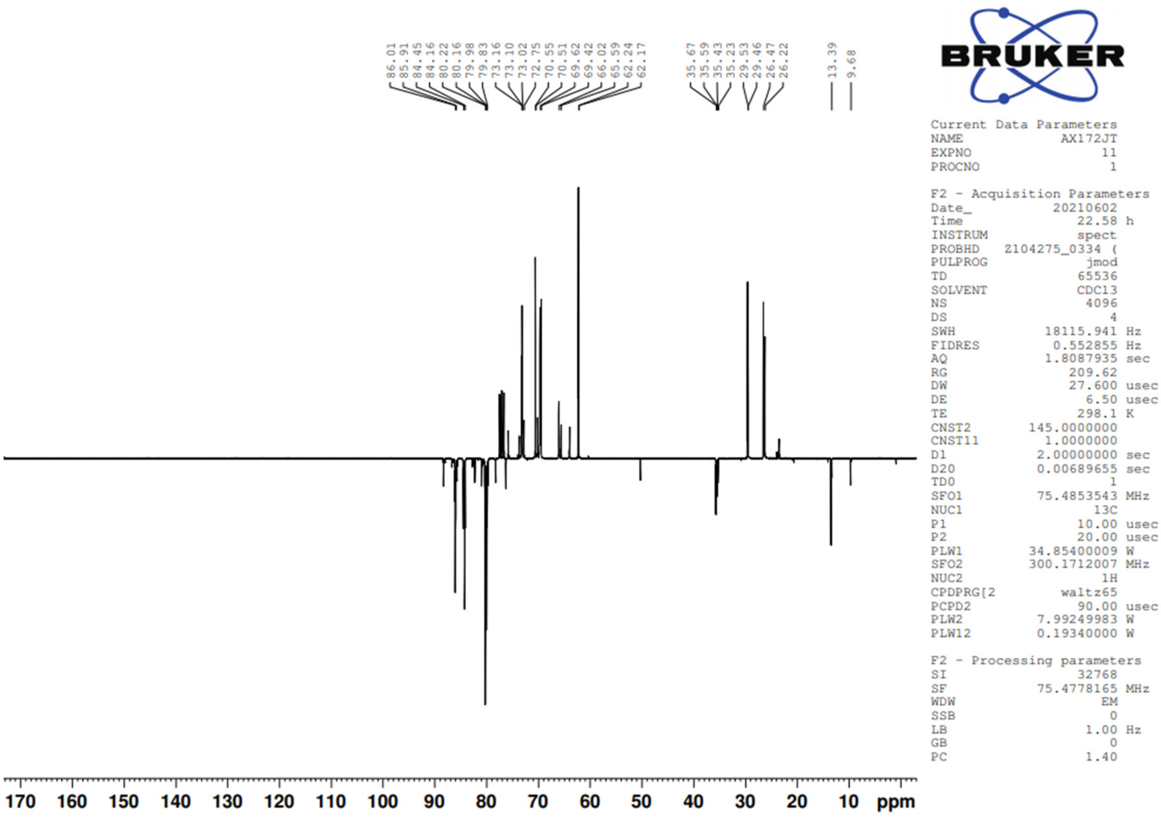

Figure S22. ^{13}C-JMOD NMR spectrum (75 MHz, $CDCl_3$, 25 °C) of hydrohydroxymethylated IDE; **R = (4-HB) or (2-HMP).**

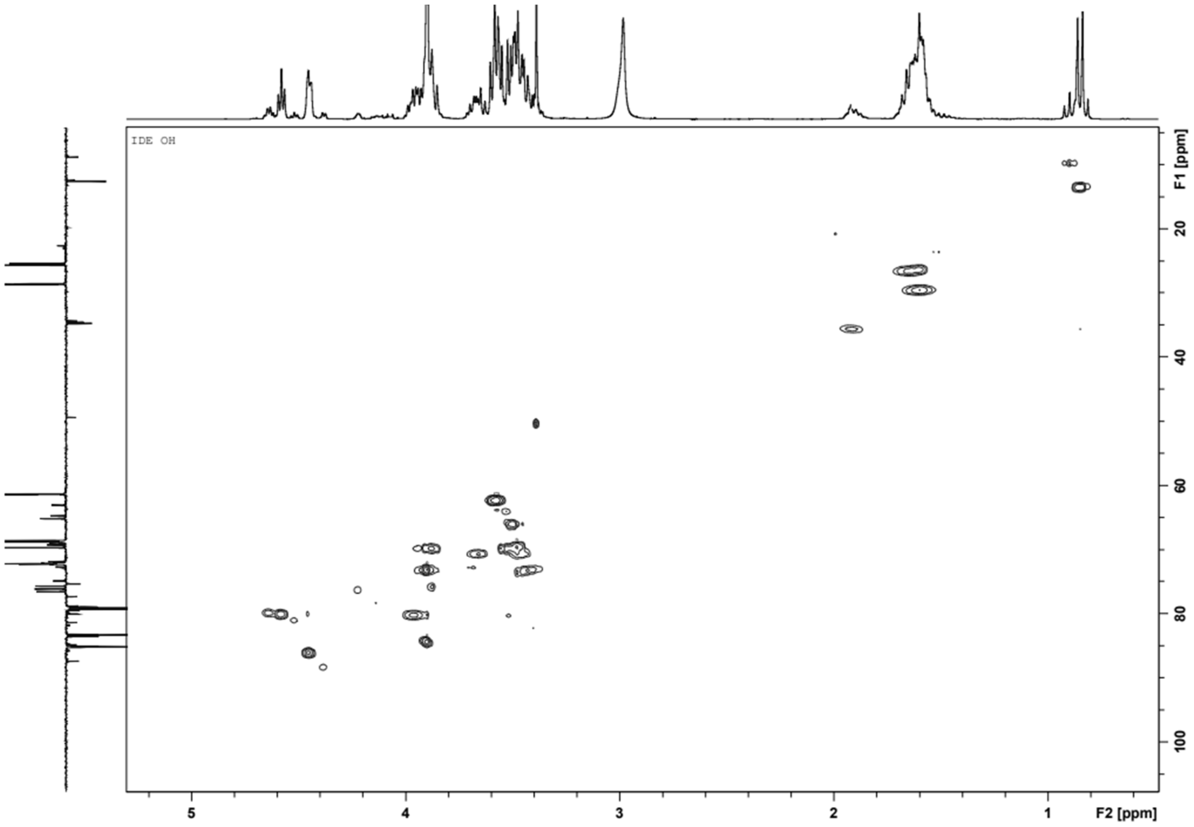

Figure S23. HSQC NMR spectrum (300 MHz, $CDCl_3$, 25 °C) of hydrohydroxymethylated IDE; **R = (4-HB) or (2-HMP).**

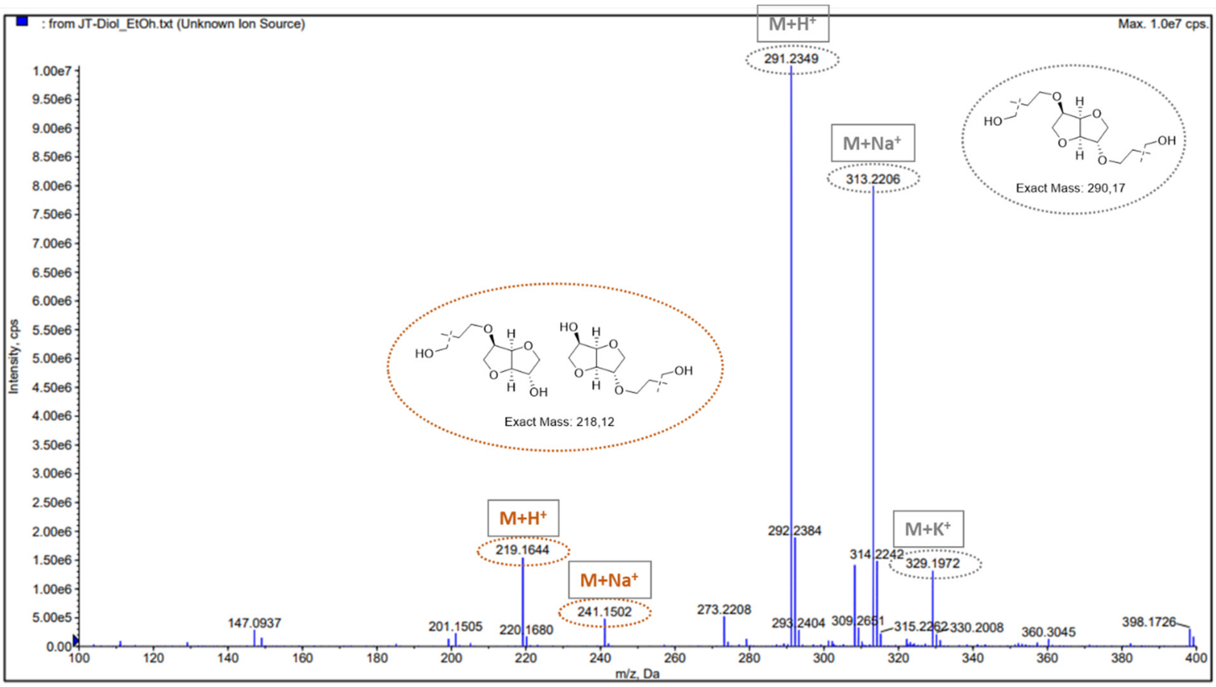

Figure S24. ESI mass spectrum of hydrohydroxymethylated IDE showing mono- and bis-alcohol products; **R = (4-HB) or (2-HMP).**

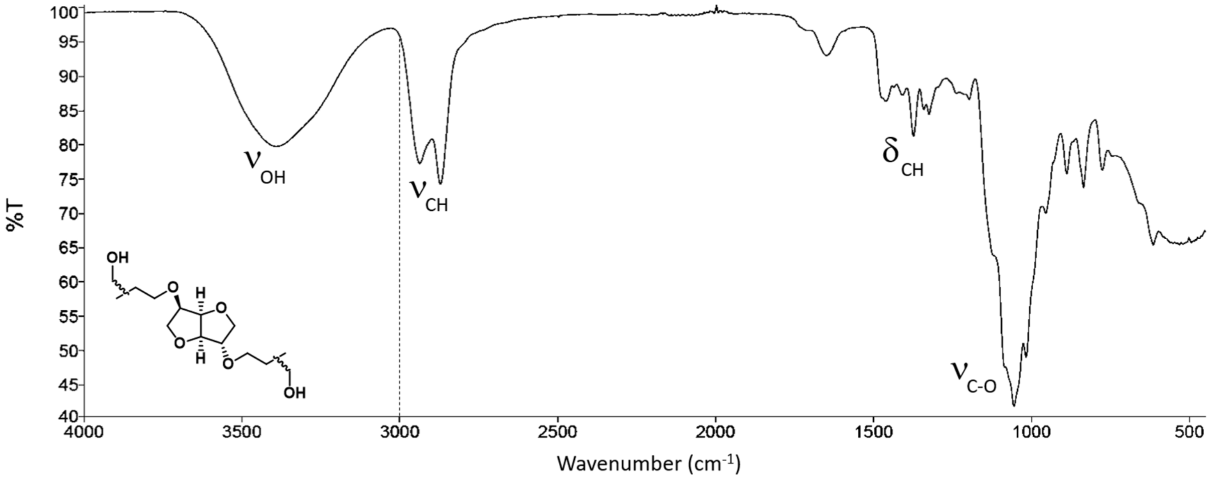

Figure S25. IR spectrum of hydrohydroxymethylated IDE**.**

IV) Determination of conversion, yields, and l/b ratios for the catalytic experiments

IV.1 Preliminary purpose

As shown with isosorbide bis-1-propenyl ether taken as substrate, once isomerized into 1-propenyl groups, allyl groups present no more reactivity in the experimental conditions used by yourself. Consequently, **(1-FP)** and **(1-HMP)** groups cannot be obtained from isosorbide diallyl ether (IDE), limiting the different possible products at a number of 120 (scheme 1).

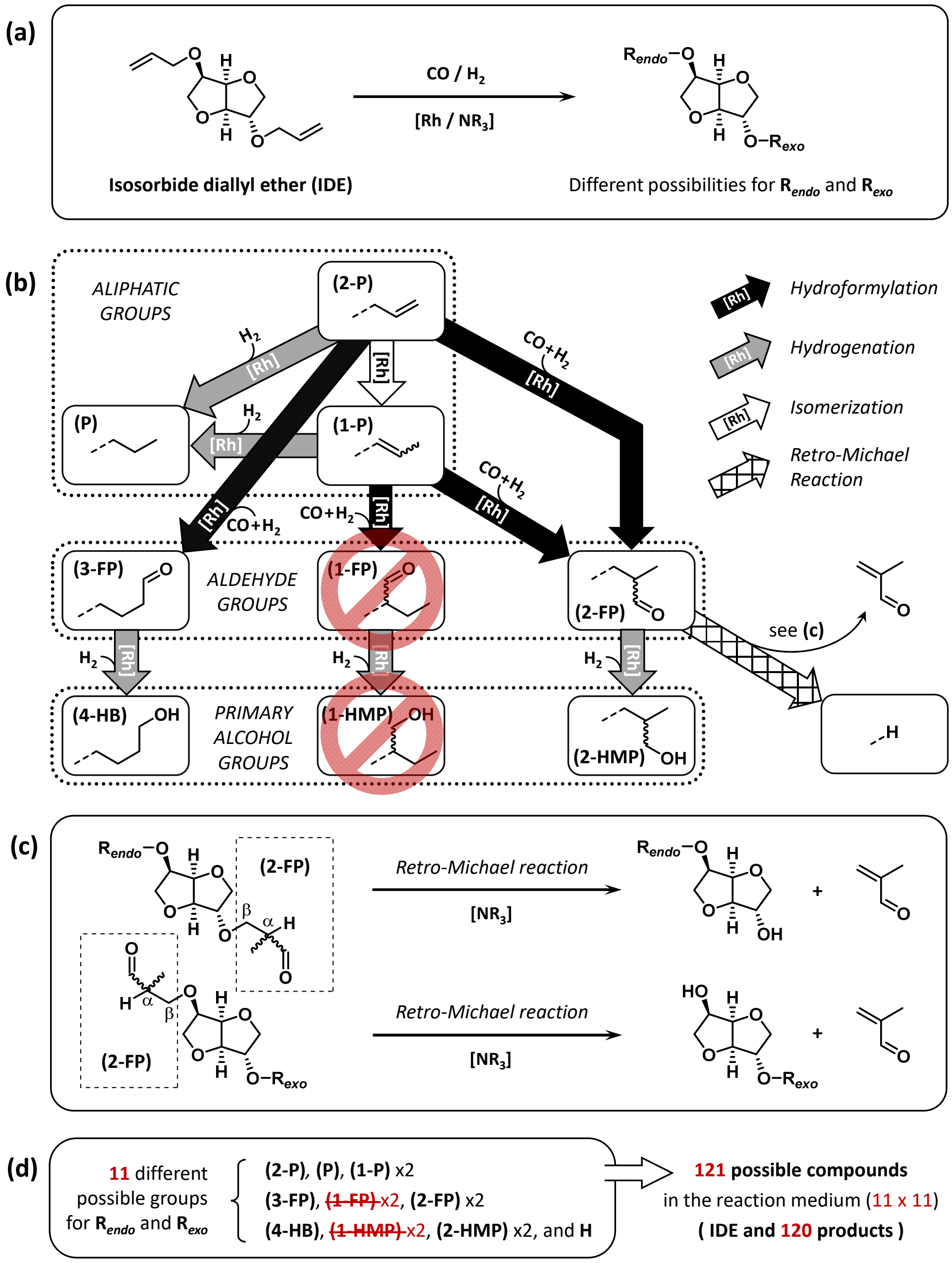

Scheme S1. Reactions of isosorbide diallyl ether (IDE) in the presence of the [Rh(acac)(CO)2/NR3] catalytic system and under CO/H2 pressure (**(1-FP)** and **(1-HMP)** groups were not formed). (a) General equation; (b) different possible groups linked to the isosorbide moiety; (c) two possible retro-Michael reactions; and (d) determination of the total number of possible compounds in the reaction medium.

In order to confirm the non-reactivity of the isomerized product bearing **(1-P)** groups (isosorbide bis(1-propenyl) ether), we first synthesized this product catalyzed by a Ru/amine system (see III.2). Once the product was formed, we used it as a starting substrate with our Rh/amine catalytic system. Figure 26 shows a superposition of the ^{1}H NMR spectra of (a) the IDE starting product, (b) of the isomerized product formed from the Ru/amine system, and (c) of the product obtained from the Rh/amine catalyzed isomerized compound. It seems clear that the isomerized product is non-reactive.

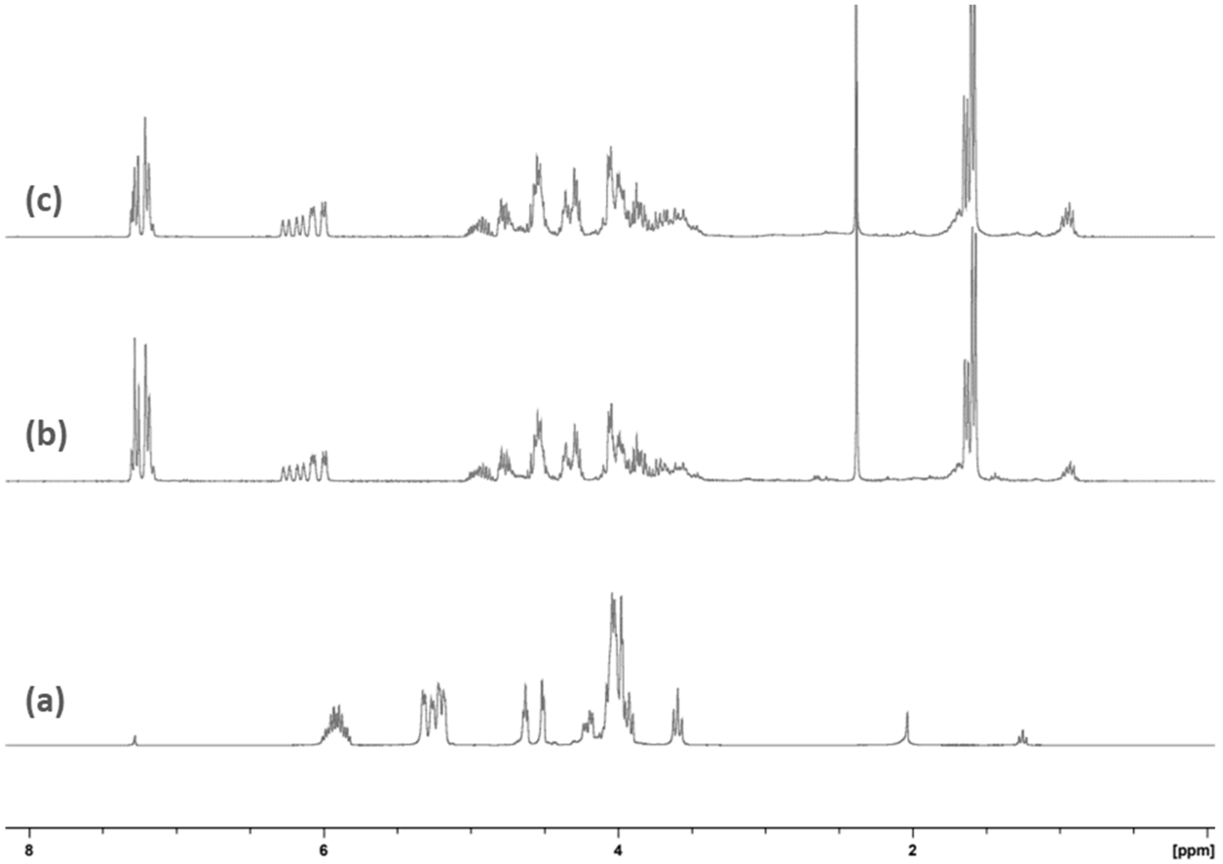

Figure S26. ^{1}H NMR spectra (300 MHz, $CDCl_3$, 25 °C) of (**a**) isosorbide diallyl ether (IDE), (**b**) isosorbide bis(1-propenyl) ether obtained from IDE with the Ru/amine system (see III.2), and (**c**) product obtained by putting the isosorbide bis(1-propenyl) ether under CO/H_2 in the presence of the Rh/amine system (experimental conditions: $Rh(acac)(CO)_2$ (12.9 mg, 0.05 mmol, 1 eq.), NEt_3 (1.012 g, 10 mmol, 200 eq.), isosorbide bis(1-propenyl) ether (2.261 g, 10 mmol, 200 eq.), toluene (6 mL), and 80 bars of CO/H_2 (1:1), 80°C, 4 h).

IV.2 Determination of conversion, yields, and l/b ratios from ^{1}H NMR spectra

Figure S27 presents a typical spectrum of the mixture of products obtained after the reaction. The chemical shift zones that have been used for conversion and yield determination are described in Table S1.

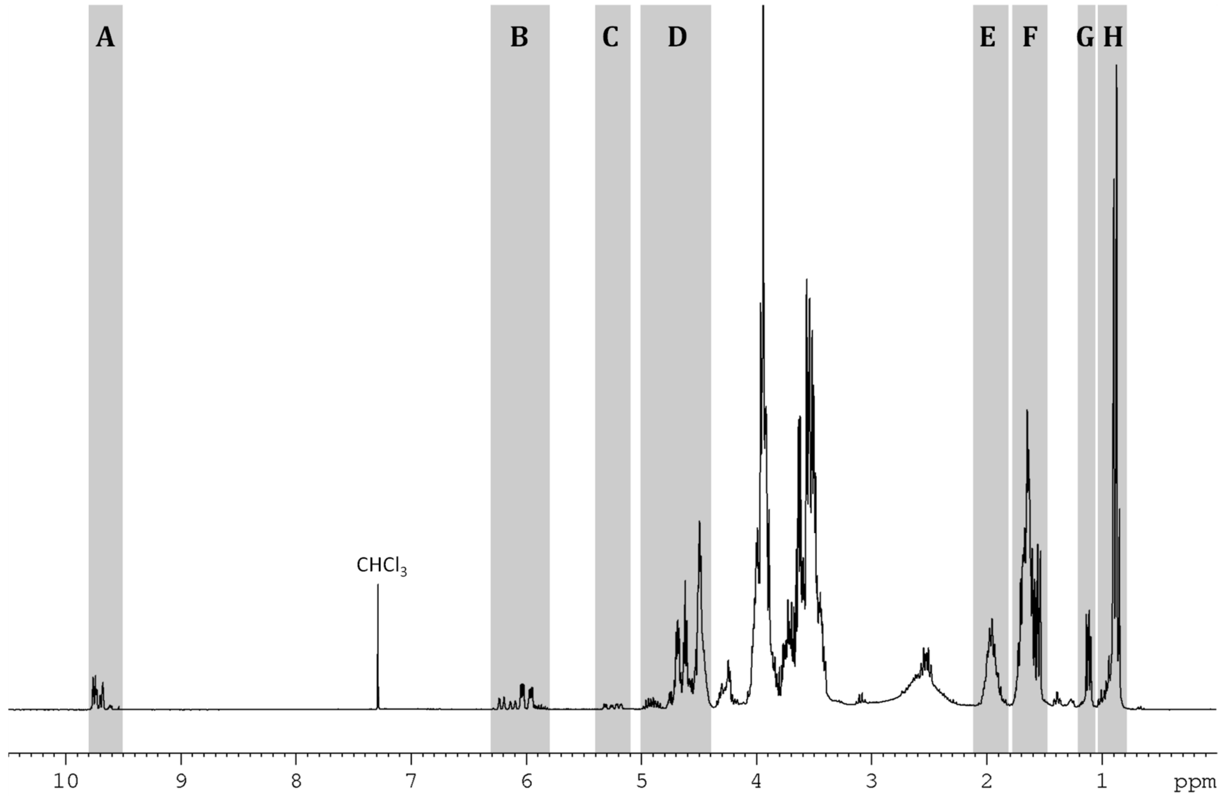

Figure S27. Typical ^{1}H NMR spectrum of the final reaction medium of isosorbide diallyl ether (IDE) hydrohydroxymethylation after evaporation of triethylamine and toluene (300 MHz, $CDCl_3$, 25 °C); the values of integration of different zones (**A** to **H**) were used to determine conversion and yields (see Table 1 for signals attributions).

From Table S1, we can deduce the eight following equations, where $\mathbf{N_{(isosorbide)}}$ and $\mathbf{N_{(X)}}$ are respectively the numbers of isosorbide moieties and **(X)** moieties in the analyzed sample, in arbitrary unit.

$$\mathbf{A = N_{(2\text{-}FP)} + N_{(3\text{-}FP)}} \quad (1)$$
$$\mathbf{B = N_{(2\text{-}P)} + N_{(1\text{-}P)}} \quad (2)$$
$$\mathbf{C = 2\ N_{(2\text{-}P)}} \quad (3)$$
$$\mathbf{D = 2\ N_{(isosorbide)} + N_{(1\text{-}P)}} \quad (4)$$
$$\mathbf{E = 2\ N_{(3\text{-}FP)} + N_{(2\text{-}HMP)}} \quad (5)$$
$$\mathbf{F = 4\ N_{(4\text{-}HB)} + 2\ N_{(P)} + 3\ N_{(1\text{-}P)}} \quad (6)$$
$$\mathbf{G = 3\ N_{(2\text{-}FP)}} \quad (7)$$
$$\mathbf{H = 3\ N_{(2\text{-}HMP)} + 3\ N_{(P)}} \quad (8)$$

Copyright: © 2021 by the authors. Licensee MDPI, Basel, Switzerland. This article is an open access article distributed under the terms and conditions of the Creative Commons Attribution (CC BY) license (https://creativecommons.org/licenses/by/4.0/).

Table S1. Denomination of the different integration values used to determine conversion and yields from the ^{1}H NMR spectrum of the concentrated reaction medium of hydrohydroxymethylation of **IDE**.

^{1}H NMR signals (ppm)	Value of integration	Involved moieties	Involved hydrogens		
9.50-9.8	**A**	**(2-FP), (3-FP)**	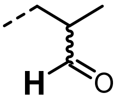	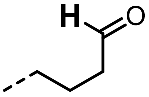	
5.80-6.30	**B**	**(2-P), (1-P)**	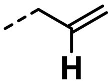	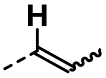	
5.10-5.40	**C**	**(2-P)**	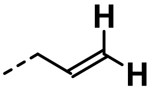		
4.40-5.00	**D**	**Isosorbide, (1-P)**	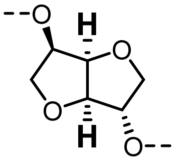	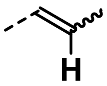	
1.80-2.10	**E**	**(3-FP), (2-HMP)**	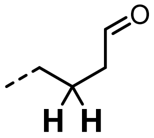	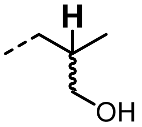	
1.50-1.80	**F**	**(4-HB), (P), (1-P)**	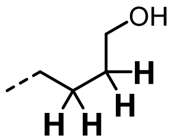	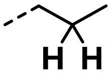	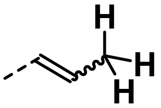
1.05-1.20	**G**	**(2-FP)**	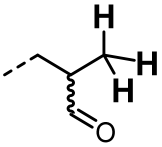		
0.80-1.05	**H**	**(2-HMP), (P)**	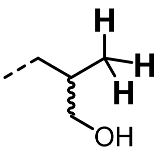	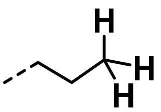	

The total number of aldehydes can be extracted from equation (1):

$$\mathbf{N_{(aldehydes)} = A} \tag{9}$$

The number of allyl groups can be calculated from equation (3):

$$\mathbf{N_{(2\text{-}P)} = C/2} \tag{10}$$

By putting equation (10) into equation (2), we obtain the number of 1-propenyl groups:

$$N_{(1\text{-}P)} = B - C/2 \quad (11)$$

By putting equation (11) into equation (4), we obtain the number of isosorbide moieties:

$$N_{(isosorbide)} = D/2 - B/2 + C/4 \quad (12)$$

The number of branched aldehydes can be extracted from equation (7):

$$N_{(2\text{-}FP)} = G/3 \quad (13)$$

The difference "(9) - (13)" gives the number of linear aldehydes:

$$N_{(3\text{-}FP)} = A - G/3 \quad (14)$$

The ratio "(14) / (13)" gives the l/b ratio for the formed aldehydes:

$$l/b_{(aldehydes)} = 3\,A/G - 1 \quad (15)$$

By putting equation (14) into equation (5), we obtain the number of branched alcohols:

$$N_{(2\text{-}HMP)} = E - 2\,A + 2\,G/3 \quad (16)$$

Equation (16) in equation (8) gives the number of propyl groups:

$$N_{(P)} = H/3 - E + 2\,A - 2\,G/3 \quad (17)$$

By putting equations (17) and (11) into equation (6), we obtain the number of linear alcohols:

$$N_{(4\text{-}HB)} = F/4 - 3\,B/4 + 3\,C/8 - H/6 + E/2 - A + G/3 \quad (18)$$

The ratio "(18) / (16)" gives the l/b ratio for the formed primary alcohols:

$$l/b_{(primary\ alcohols)} = \frac{F/4 - 3\,B/4 + 3\,C/8 - H/6 + E/2 - A + G/3}{E - 2\,A + 2\,G/3} \quad (19)$$

The sum "(16) + (18)" gives the total number of primary alcohols:

$$N_{(primary\ alcohols)} = 3\,E/2 - 3\,A + G + F/4 - 3\,B/4 + 3\,C/8 - H/6 \quad (20)$$

Naming $N_{(RM)}$ the number of initial allyl groups initially bound to the isosorbide that have been transformed into methacrolein by retro-Michael reaction and before possible further reactions, we can write, by mass conservation:

$$2\,N_{(isosorbide)} = N_{(RM)} + N_{(2\text{-}P)} + N_{(1\text{-}P)} + N_{(P)} + N_{(2\text{-}FP)} + N_{(3\text{-}FP)} + N_{(2\text{-}HMP)} + N_{(4\text{-}HB)} \quad (21)$$

So, from equation (21), we obtain:

$$N_{(RM)} = 2\,N_{(isosorbide)} - N_{(2\text{-}P)} - N_{(1\text{-}P)} - N_{(P)} - N_{(2\text{-}FP)} - N_{(3\text{-}FP)} - N_{(2\text{-}HMP)} - N_{(4\text{-}HB)} \quad (22)$$

By putting equations (10), (11), (12), (13), (14), (16), (17), and (18) into equation (22), we obtain:

$$N_{(RM)} = D - 5\,B/4 + C/8 - H/6 - E/2 - G/3 - F/4 \quad (23)$$

Note that $N_{(RM)}$ corresponds to the number of secondary hydroxyl groups that have appeared on the isosorbide moieties due to the retro-Michael reactions of (**2-FP**) groups.

Conversion of the allyl group can be written in the following way:

$$\mathbf{Conv.}\,(\%) = \frac{N_{(RM)} + N_{(1.P)} + N_{(P)} + N_{(2.FP)} + N_{(3.FP)} + N_{(2.HMP)} + N_{(4.HB)}}{2\,N_{(isosorbide)}} \times 100 \quad (24)$$

or, more simply, by using the equation (21):

$$\mathbf{Conv.}\,(\%) = \frac{2\,N_{(isosorbide)} - N_{(2.P)}}{2\,N_{(isosorbide)}} \times 100 \quad (25)$$

Thanks to equations (10) and (12), we obtain:

$$\mathbf{Conv.}(\%) = \frac{\mathbf{D - B}}{\mathbf{D - B + C/2}} \times \mathbf{100} \quad (26)$$

For each (**X**) graft on the isosorbide moieties, including the hydrogen atom due to the retro-Michael reaction, the corresponding yield is defined by equation (27).

$$\mathbf{Y_{(X)}}(\%) = \frac{\mathbf{N_{(X)}}}{\mathbf{2\ N_{(isosorbide)}}} \times \mathbf{100} \quad (27)$$

Using equation (12) and, respectively, the equations (11), (17), (9), (20), and (23), the different yields can be determined from the ^{1}H NMR spectrum integration values:

$$\mathbf{Y_{(1\text{-}P)}}(\%) = \frac{\mathbf{B - C/2}}{\mathbf{D - B + C/2}} \times \mathbf{100} \quad (28)$$

$$\mathbf{Y_{(P)}}(\%) = \frac{\mathbf{H/3 - E + 2\ A - 2\ G/3}}{\mathbf{D - B + C/2}} \times \mathbf{100} \quad (29)$$

$$\mathbf{Y_{(aldehydes)}}(\%) = \frac{\mathbf{A}}{\mathbf{D - B + C/2}} \times \mathbf{100} \quad (30)$$

$$\mathbf{Y_{(primary\ alcohols)}}(\%) = \frac{\mathbf{3\ E/2 - 3\ A + G + F/4 - 3\ B/4 + 3\ C/8 - H/6}}{\mathbf{D - B + C/2}} \times \mathbf{100} \quad (31)$$

$$\mathbf{Y_{(RM)}}(\%) = \frac{\mathbf{D - 5\ B/4 + C/8 - H/6 - E/2 - G/3 - F/4}}{\mathbf{D - B + C/2}} \times \mathbf{100} \quad (32)$$

To resume, after integration of the ^{1}H NMR spectrum and determination of the different integration values **A**, **B**, **C**, **D**, **E**, **F**, **G**, and **H**, the allyl groups conversion ($\mathbf{Conv.}(\%)$), the yields with respect to the initial allyl group ($\mathbf{Y_{(X)}}(\%)$), and the $\boldsymbol{l/b}$ ratio for the formed aldehydes and primary alcohols can be determined by the following expressions.

$$\mathbf{Conv.}(\%) = \frac{\mathbf{D - B}}{\mathbf{D - B + C/2}} \times \mathbf{100} \quad (26)$$

$$\mathbf{Y_{(P)}}(\%) = \frac{\mathbf{H/3 - E + 2\ A - 2\ G/3}}{\mathbf{D - B + C/2}} \times \mathbf{100} \quad (29)$$

$$\mathbf{Y_{(1\text{-}P)}}(\%) = \frac{\mathbf{B - C/2}}{\mathbf{D - B + C/2}} \times \mathbf{100} \quad (28)$$

$$\mathbf{Y_{(aldehydes)}}(\%) = \frac{\mathbf{A}}{\mathbf{D - B + C/2}} \times \mathbf{100} \quad (30)$$

$$\mathbf{Y_{(primary\ alcohols)}}(\%) = \frac{\mathbf{3\ E/2 - 3\ A + G + F/4 - 3\ B/4 + 3\ C/8 - H/6}}{\mathbf{D - B + C/2}} \times \mathbf{100} \quad (31)$$

$$\mathbf{Y_{(RM)}}(\%) = \frac{\mathbf{D - 5\ B/4 + C/8 - H/6 - E/2 - G/3 - F/4}}{\mathbf{D - B + C/2}} \times \mathbf{100} \quad (32)$$

$$\boldsymbol{l/b}_{\mathbf{(aldehydes)}} = \mathbf{3\ A/G\ - 1} \quad (15)$$

$$\boldsymbol{l/b}_{\mathbf{(primary\ alcohols)}} = \frac{\mathbf{F/4 - 3\ B/4 + 3\ C/8 - H/6 + E/2 - A + G/3}}{\mathbf{E - 2\ A + 2\ G/3}} \quad (19)$$

For a better understanding, l/b ratios were transformed into $l(\%)$ and $b(\%)$ relative percentages, thanks to the two following relations:

$$l(\%) = \frac{l/b}{l/b+1} \times 100 \qquad b(\%) = \frac{100}{l/b+1} \qquad (33)$$

IV.3 Determination of yield (Y'$_{(BPA)}$) and selectivity ratios ($[ll/lb/bb]$) for the bis-primary alcohols from GC-FID chromatograms

Figure 28 presents a typical GC-FID chromatogram of a reaction mixture obtained after a catalytic test involving isosorbide diallyl ether as a substrate. The retention time of each compound type was determined by GC analyses of authentic samples.

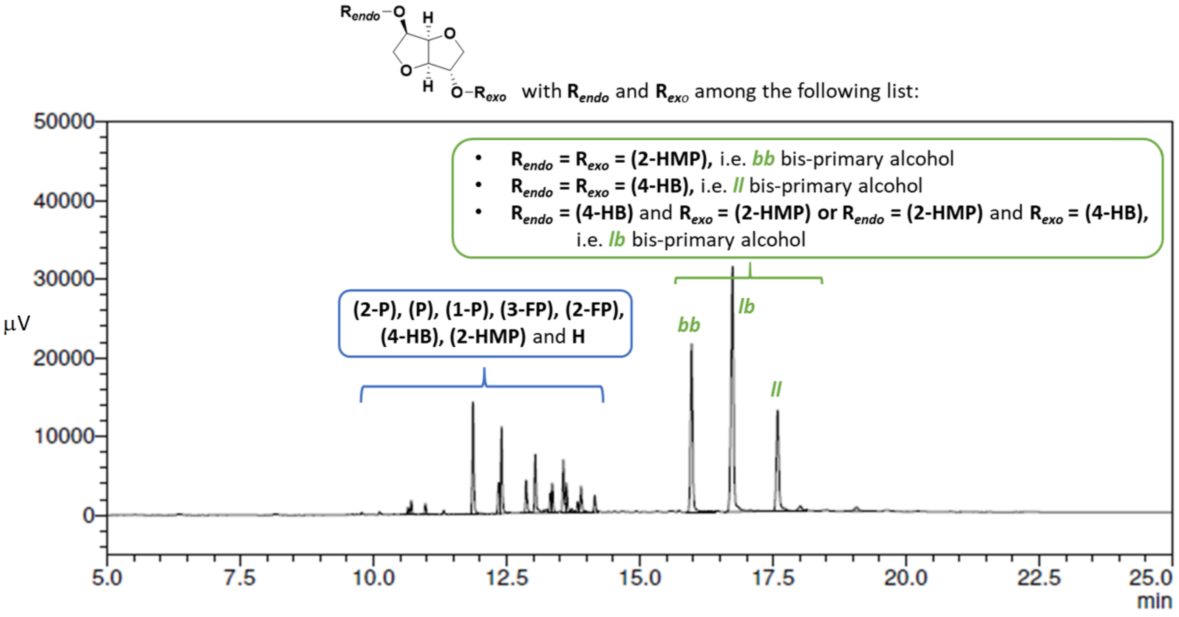

Figure S28. Chromatogram of a reaction mixture obtained after a catalytic test; conditions: initial temperature of 50 °C, increased to 250 °C by 15 °C/min and held for 15 min. The injector and detector temperatures were 250 °C and nitrogen was used as the carrier gas at a constant column flow rate of 1.50 mL/min. An aliquot of the sample was injected in split mode.

Briefly, polar bis-primary alcohols (**BPA**) possess the highest retention times (between 15.9 and 17.6 minutes), while mono-primary alcohols, other less polar reaction products, and substrates (IDE) give peaks at retention times ranging from 10.7 to 14.5 minutes.

So, by naming $\mathbf{S}_{(BPA)}$ the sum of the areas of the chromatographic peaks between 15.9 and 17.6 minutes and $\mathbf{S}_{(others)}$ the sum of the areas of the chromatographic peaks between 10.6 and 14.5 minutes, the GC yield in bis-primary alcohol ($\mathbf{Y'}_{(BPA)}$), with respect to the initial isosorbide moiety, can be estimated, thanks to equation (34).

$$Y'_{(BPA)}(\%) = \frac{S_{(BPA)}}{S_{(BPA)} + S_{(others)}} \times 100 \qquad (34)$$

$$[ll\ /\ lb\ /\ bb] = [\frac{S_{(ll)}}{S_{(BPA)}} \times 100\ /\ \frac{S_{(lb)}}{S_{(BPA)}} \times 100\ /\ \frac{S_{(bb)}}{S_{(BPA)}} \times 100\ ] \qquad (35)$$

$$S_{(BPA)} = S_{(ll)} + S_{(lb)} + S_{(ll)} \qquad (36)$$

Moreover, the chromatographic peaks between 15.9 and 17.6 minutes are attributed to ***bb*-**, ***lb*-**, and ***ll*-**bis-primary alcohols, in this order. So, by naming $S_{(bb)}$, $S_{(lb)}$, and $S_{(ll)}$, the corresponding chromatographic areas of these three peaks, the $[ll/lb/bb]$ selectivity ratios for the bis-primary alcohols are estimated, thanks to equations (35) and (36).

IV.4 Kinetic follow-up of an isosorbide diallyl ether hydrohydroxymethylation catalytic test

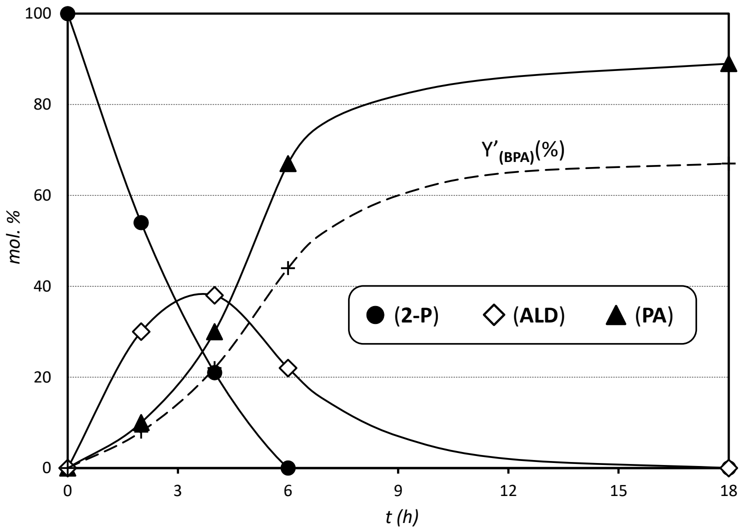

Figure S29. Kinetic follow-up of an isosorbide diallyl ether hydrohydroxymethylation catalytic test. Experimental conditions: $Rh(acac)(CO)_2$ (12.9 mg, 50 µmol, 1 equiv), IDE (2.26 g, 10 mmol, 200 equiv), TEA (20 equiv), toluene (6 mL), and 80 bars of CO/H_2 (1:1), 80 °C. The mol. % of **(2-P)** groups (2-propenyl), **(ALD)** groups (aldehyde groups = **(2-FP)** + **(3-FP)**), and **(PA)** groups (primary alcohol groups = **(2-HMP)** + **(4-HB)**) were determined by ^1H NMR. For more clarity, mol. % of **(P)** groups (propyl), **(1-P)** group (1-propenyl), and **(RM)** groups (= **(2-FP)** grafts that have been cleaved by retro-Michael reaction) were not reported; indeed, all these mol. % are inferior or equal to 5. $\mathbf{Y'_{(BPA)}}$ = yield in bis-primary alcohols with respect to the initial isosorbide moiety, determined by gas chromatography (GC-FID).
